# Tumor necrosis factor receptor 2 promotes endothelial cell-mediated suppression of CD8+ T cells through tuning glycolysis in chemoresistance of breast cancer

**DOI:** 10.1186/s12967-024-05472-5

**Published:** 2024-07-20

**Authors:** Yu Hu, Xiaohan Lou, Kaili Zhang, Longze Pan, Yueyue Bai, Linlin Wang, Ming Wang, Yan Yan, Jiajia Wan, Xiaohan Yao, Xixi Duan, Chen Ni, Zhihai Qin

**Affiliations:** 1 Henan China–Germany International Joint Laboratory of Tumor Immune Microenvironment and Disease, Medical Research Center, The First Affiliated Hospital of Zhengzhou University, Zhengzhou, 450052 Henan China; 2 Department of Medicine, Luohe Medical College, Luohe, 462000 China; 3 Shangqiu Hospital, The First Affiliated Hospital of Henan University of Chinese Medicine, Shangqiu, 476000 China

**Keywords:** Triple negative breast cancer, Paclitaxel, Chemoresistance, Endothelial cells, CD8+ T cells, TNFR2

## Abstract

**Background:**

T cells play a pivotal role in chemotherapy-triggered anti-tumor effects. Emerging evidence underscores the link between impaired anti-tumor immune responses and resistance to paclitaxel therapy in triple-negative breast cancer (TNBC). Tumor-related endothelial cells (ECs) have potential immunoregulatory activity. However, how ECs regulate T cell activity during TNBC chemotherapy remains poorly understood.

**Methods:**

Single-cell analysis of ECs in patients with TNBC receiving paclitaxel therapy was performed using an accessible single-cell RNA sequencing (scRNA-seq) dataset to identify key EC subtypes and their immune characteristics. An integrated analysis of a tumor-bearing mouse model, immunofluorescence, and a spatial transcriptome dataset revealed the spatial relationship between ECs, especially Tumor necrosis factor receptor (TNFR) 2+ ECs, and CD8+ T cells. RNA sequencing, CD8+ T cell proliferation assays, flow cytometry, and bioinformatic analyses were performed to explore the immunosuppressive function of TNFR2 in ECs. The downstream metabolic mechanism of TNFR2 was further investigated using RNA sequencing, cellular glycolysis assays, and western blotting.

**Results:**

In this study, we identified an immunoregulatory EC subtype, characterized by enhanced TNFR2 expression in non-responders. By a mouse model of TNBC, we revealed a dynamic reduction in the proportion of the CD8+ T cell-contacting tumor vessels that could co-localize spatially with CD8+ T cells during chemotherapy and an increased expression of TNFR2 by ECs. TNFR2 suppresses glycolytic activity in ECs by activating NF-κB signaling in vitro. Tuning endothelial glycolysis enhances programmed death-ligand (PD-L) 1-dependent inhibitory capacity, thereby inducing CD8+ T cell suppression. In addition, TNFR2+ ECs showed a greater spatial affinity for exhausted CD8+ T cells than for non-exhausted CD8+ T cells. TNFR2 blockade restores impaired anti-tumor immunity in vivo, leading to the loss of PD-L1 expression by ECs and enhancement of CD8+ T cell infiltration into the tumors.

**Conclusions:**

These findings reveal the suppression of CD8+ T cells by ECs in chemoresistance and indicate the critical role of TNFR2 in driving the immunosuppressive capacity of ECs via tuning glycolysis. Targeting endothelial TNFR2 may serve as a potent strategy for treating TNBC with paclitaxel.

**Supplementary Information:**

The online version contains supplementary material available at 10.1186/s12967-024-05472-5.

## Introduction

Paclitaxel (PTX) is acutely cytotoxic and has long been the first-line therapeutic option for triple-negative breast cancer (TNBC) [1]. PTX-triggered immunogenic cell death (ICD) of tumor cells elicits a massive infiltration of tumor-reactive CD8+ T cells [2], which are considered central to anti-tumor effects and can act as a well-characterized biomarker for efficacy [3, 4]. It has been documented that the endothelium serves as an ineluctable interface involved in T cell homing to tumor cores; ECs not only direct T cell trafficking, but also reshape T cell function and differentiation [5–7]. Due to the huge heterogeneity of ECs [8, 9], it is not appropriate to combine them as a single population. Certain EC subtypes reportedly contribute to angiogenesis and extracellular matrix remodeling in breast cancer, whereas another subtype is involved in immunomodulation [9]. The identification of the specific EC subtypes involved in chemoresistance is urgently required and is made possible by single-cell RNA sequencing. As the cellular landscape of tumor ecosystems is polarized by the specific efficacy of chemotherapy, it remains still difficult to better characterize endothelial diversity, especially in chemotherapeutic settings.

T cell interactions with neighboring antigen-presenting cells (APCs), including ECs, are contact-dependent. Determining the mechanism by which ECs regulate T cell function during chemotherapy is of great significance. For instance, we previously determined that IFNγ-dependent angiostasis is a key mechanism in T cell-mediated anti-tumor immunity [10, 11]. A feedback phenomenon occurs where IFNγ triggers upregulation of inhibitory molecules, like PD-L1 [12] and indoleamine 2,3-dioxygenase (IDO) 1 [13], potentially acting as an immune-excluded mechanism of ECs. The spatial distance between ECs and T cells limits their interactions, notably through a feedback mechanism. For TNBC, whether and to what extent ECs are spatially close to T cells and the dynamics of their spatial relationship during chemotherapy remain to be determined.

Inflammatory mediators in the tumor microenvironment (TME) determine the type of cancer-related immune reaction generated. The pro-inflammatory cytokine tumor necrosis factor (TNF)α is considered a key regulator and acts by binding to one of two distinct functional surface receptors [14], TNF receptor (TNFR)1 and TNFR2. Our previous studies demonstrated that TNFR2, but not TNFR1, is immunosuppressive [15, 16]. It was reported that TNFα can promote endothelial PD-L1 expression in response to IFNγ [17], but the receptor involved remains unknown. Therefore, we hypothesize that TNFR2 in cancer-related ECs might be involved in the suppression of CD8+ T cells and resistance to PTX agent.

The modulation of energy metabolism contributes to the adaptation of cells to harsh living environments, as exemplified by rewiring the hyperglycolytic catabolism of ECs exposed to external stress [18]. Exploring the changes in glycolytic metabolism and identifying the molecular mechanisms of ECs related to their immunosuppressive capacity take on special significance. Previous studies have shown that the generation and maintenance of PD-L1 depend on glucose metabolism [19] in tumor cells and lactate [20] in macrophages. We investigated whether the regulation of glycolytic activity in ECs contributes to endothelial immunosuppression.

The present study used integrated analyses of publicly accessible multi-omic datasets, RNA sequencing, and a series of experiments in vitro and in vivo to identify the immunoregulatory phenotype of ECs with enhanced TNFR2 expression related to tumor resistance to PTX therapy. TNFR2-turning glycolysis in ECs further promotes PD-L1 expression. TNFR2+ ECs mediate the exhaustion of CD8+ T cells via the PD-L1/Programmed cell death (PD)-1 pathway and are spatially close to the latter. Our study provides comprehensive insight into the immune features and dynamics of ECs in chemotherapeutic settings.

## Methods and materials

### Main reagents

Paclitaxel (P815862, MACKLIN) was dissolved in a mixture of corn oil and ethanol. Human TNFα (AF-300-01A), murine TNFα (AF-315-01A) and murine IFNγ (AF-315-05) were purchased from Peprotech. Anti-TNFR2 antibody (TR75-54.7, Bio X Cell) was used for the in vivo experiments. Anti-TNFR1 (113103, BioLegend) and TNFR2 (113305, BioLegend) antibodies were prepared for the in vitro experiments. SC75741 (S7273, Selleck) is a potent inhibitor for NF-κB activation.

### Cell and culture conditions

sEND.1 cell (RRID: CVCL_6270) is a sort of immortalized vascular endothelial cell from skin angioma and was established in Dr. Blankenstein’s laboratory [21]. Human umbilical vein endothelial cell (HUVEC; RRID: CVCL_2959) and the mouse triple-negative breast cancer cell line 4T1 (RRID: CVCL_0125) were purchased from ATCC (Manassas, VA). These cells were cultured in mycoplasma-free Dulbecco’s modified Eagle’s medium (DMEM; SH10022.01, Hyclone), supplemented with 10% FBS (P30-3302, PAN) and 1% penicillin/streptomycin (SV30010, Hyclone).

### Tumor model and treatment

Sex-matched (female) and age-matched (6–8 weeks old) BABL/c wild-type (WT) mice were purchased from Vital River Laboratories (Beijing, China) and randomized to the different experimental groups. All the mice were maintained under specific pathogen-free conditions. 4T1 cells (0.5 × 10^6^/per) in 100 μl PBS were subcutaneously (s.c.) injected into breast pad of mice. Tumor growth was monitored every 2–3 d during the entire experimental period. Tumor volumes (V) were assessed as follows: V = 0.5 × (L × W)^2^. L represents the long diameter of the tumor mass and W represents the short diameter. Starting on day 5–7 post inoculation, paclitaxel (20 mg/kg) agent, Anti-TNFR2 (10 mg/kg) antibodies, or PBS as a control were intraperitoneally (i.p.) injected into the mice when the tumor volume reached approximately 100 mm^3^. Additional treatment scheduling is shown in Figs. 2A and 8A.

### RNA-sequencing analysis

The total RNA of sEND.1 cells administrated with co-stimulation of TNF (20 ng/ml) and IFNγ (10 ng/ml), or IFNγ (10 ng/ml) as control for 24 h, was extracted by mRNA isolation kit V2 (L/N 7E522J1, Vazyme, China) and then RNA sequencing was performed by BGI Genomics (Shenzhen, China). Differentially expressed genes (DEGs) were defined at the settled condition of P_adjust < 0.05 and a log2 fold change (FC) > 0.5. Subsequent data from RNA-sequencing (RNA-seq), such as volcano plots, heatmaps, Kyoto Encyclopedia of Genes and Genomes (KEGG) and Gene set enrichment analysis (GSEA) were obtained from Dr. Tom’s website created by BGI.

### Flow cytometry

The expression of PD-L1 or TNFR2 on EC was detected using flow cytometry (FCM). These single-cell suspensions were prepared in vitro or from tumor tissues and then stained with antibodies specific for CD45− Percp-cy5.5 (157612, BioLegend), CD31-FITC (160212, BioLegend), PD-L1-PE (12-5982-82, eBioscience), mouse TNFR2-PE (550086, BD) and human TNFR2-PE (552418, BD). Similarly, suspensions for CD8+ T cells isolated from tumor tissues were stained with antibodies specific for CD45-Percp-cy5.5, CD3-FITC (100203, BioLegend), CD8-APC (100712, BioLegend) and PD-1-PE (135205, BioLegend). Cell suspensions were performed using FACS Calibur devic (BD, USA) and raw data was analyzed using Flow Jo software (RRID:SCR_008520; BD, USA).

### Western blotting

sEND.1 cells were harvested and lysed with RIPA buffer (R0020, Solarbio), supplemented with PMSF (P8340-1, Solarbio) and a protease inhibitor cocktail (P8340, Sigma). Cell lysates were collected in RIPA buffer and the protein concentration was quantitated by Protein BCA Assay Kits (23228, Thermo Fisher Scientific). An equal number of proteins was separated by SDS-PAGE and transferred onto nitrocellulose membranes, followed overnight incubation with rabbit anti-NF-κB antibody (65 kDa, 1:1000, 8242 s, CST), rabbit anti-pNF-κB antibody (65 kDa, 1:1000, 3033 s, CST), rabbit-anti-Glut1 (54 kDa, 1:1000, 07-1401, Millipore), rabbit-anti-HK2 (102 kDa, 1:1000, 2867 s, CST), rabbit-anti-pPFKFB2 (55 kDa, 1:1000, 13064 s, CST), mouse anti-β-actin antibody (42 kDa, 1:5000, AC004, abclonal). The nitrocellulose membranes were then incubated with horseradish peroxidase (HRP)-conjugated secondary antibodies. Protein bands were visualized using an eECL Western Blot Kit (CW00495, CwBio) and detected using a ChemiDoc MP Imaging System (Bio-Rad).

### Quantitative real-time PCR (qRT-PCR)

The total RNA was extracted from sEND.1 cells using mRNA isolation kit V2 (L/N 7E522J1, Vazyme) according to the manufacturer’s instructions, then converted into cDNA using the Prime Script™ RT reagent kit (RR047A, Takara) and subjected to subsequent qPCR using SYBR Premix Ex Taq II (RR820A, Takara). The primers for mouse mRNAs are listed as follows: *Gapdh* forward: TCTCTGCTCCTCCCTGTTCC, reverse: TACGGCCAAATCCGTTCACA; *Pd-l1* forward: TGCGGACTACAAGCGAATCACG, reverse: CTCAGCTTCTGGATAACCCTCG; Pvr forward: GAGGCAGTAGAAGCACCAATGC, reverse: GGTGACCATTGGCAGAGATGCA; *Vista* forward: AACAACGGTTCTACGGGTCC, reverse: CGTGATGCTGTCACTGTCCT; *Tigit* forward: CCACAGCAGGCACGATAGATA, reverse: CATGCCACCCCAGGTCAAC; *H2-d1* forward: TGAGGAACCTGCTCGGCTACTA, reverse: GGTCTTCGTTCAGGGCGATGTA; *H2-k1* forward: GGCAATGAGCAGAGTTTCCGAG, reverse: CCACTTCACAGCCAGAGATCAC; *H2-q1* forward: GCTGTTCTGGTTGTCCTTGGAG, reverse: AGAGCACAGTCCTCTCCTTGTC.

### Immunofluorescence staining

Tumor tissues were isolated and embedded in cutting temperature compound (OCT) and therewith prepared as cryostat sections. Frozen tissue sections (7 μm) were fixed with 4% paraformaldehyde for 15 min and permeabilized three times at room temperature. After blockade by 2% bovine serum albumin, the sections were incubated overnight with the following primary specific antibodies: rat anti-CD31 (1:200, 550274, BD), rabbit anti-TNFR2 (1:200, ab109322, Abcam) and rabbit anti-CD8 (1:400, ab217344, Abcam). Following incubation with fluorescence-conjugated secondary antibodies (1:400), the tissue sections were stained with DAPI (1:3000). Images were acquired using an inverted fluorescence microscope (Leica).

### Manders’ colocalization coefficients

To better quantify the fraction of one protein that colocalizes with another distinct protein, Manders’ Colocalization Coefficients (MCC) were created by Manders et al. [22]. In this study, we determined and quantified the dynamics of spatial relationship between ECs and CD8+ T cells using MCC analysis. M1 in an image represents the ratio of the part of CD8 protein that colocalizes with another protein (CD31) to the whole, that is, the overlapping fraction of CD8; M2 represents the same pattern of CD31. Biological image analysis of MCC performed using an auto plug-in in Fiji/Image J (RRID: SCR_003070; NIH). The formulae for calculating value of M1 and M2 in this study are as follows:

$${M_{1}} = \frac{{\sum\nolimits_{i} {{CD8}_{i,colocal} } }}{{\sum\nolimits_{i} {{CD8}_{i} } }}\quad {M_{2}} = \frac{{\sum\nolimits_{i} {{CD31}_{i,colocal} } }}{{\sum\nolimits_{i} {{CD31}_{i} } }}$$

### Glucose analog uptake

As a glucalogue, 2-[*N*-(7-Nitrobenz-2-oxa-1,3-diaxol-4-yl) amino]-2-deoxyglucose (2-NBDG) can be taken up by cells through glucose transmembrane transfer. sEND.1 cells (1 × 10^5^ cells) were seeded in 24-well plates. After treatment with or without TNFα for 6 h, cells were rewashed with PBS, followed by an addition of low glucose culture media supplemented with 100 µM 2-NBDG (N13195, Life Technologies) and incubation for 45 min at 37 °C. An additional group lacking 2-NBDG was set as blank control. Next, the cells were harvested and 2-NBDG uptake was immediately detected by flow cytometry in the distinct groups.

### Cellular glycolysis assay

The change in extracellular acidification rate (ECAR) for ECs regulated by TNFα was detected. sEND.1 cells were seeded into seahorse XF-96 microplate (6000 cells per well) for overnight, and then treated with or without TNFα (20 ng/ml) for 6 h. Culture medium was replaced with the XF base medium (pH 7.4) supplemented with 2 mM l-glutamine (V900419, Sigma-Aldrich), followed by incubated in a non-CO2 incubator for 30 min. Subsequently, glucose, oligomycin, and 2-deoxy-d-glucose (2-DG) were added into the medium at final concentration of 10 mM, 1 μM, 50 mM, respectively. Finally, ECAR was measured by Seahorse XF-96 extracellular flux analyzer (Seahorse Bioscience, Agilent) and the corresponding number of ECs was normalized by hoechst 33342 (C0030, Solarbio) according to the manufacturer’s instructions.

### CD8+ T cell proliferation assays

After filtration of grinded spleens freshly isolated from tumor-free OT-I mice (003831, Jackson Laboratory), splenocytes were incubated overnight in RPMI1640 complete medium containing IL-2 (10 ng/ml; AF-212-12, Peprotech) to remove adherable cells. The next day, splenocytes were labeled with CFSE (5 mM; 21888, Sigma-Aldrich) for 10 min and then neutralized with RPMI1640. Both splenocytes (30 × 10^4^) and sEND.1 (6 × 10^2^) were pre-treated by IFNγ (20 ng/ml) and TNFα (10 ng/ml). Anti-TNFR2 antibody (10 μg/ml) was pre-treated 1 h prior to administration of TNFα. The cell mixture in a total of 100 µl culture medium containing IL-2 (10 ng/ml), SIINFEKL (0.1 μM; S7951, Sigma-Aldrich) and anti-mouse CD3 (3 μg/ml; 550275, BD Biosciences) and CD28 antibodies (1 μg/ml; 557393, BD Biosciences) was dispensed into a well of 96-well round-bottom plates and cultured for at least 72 h at 37 °C. CD8+ T cells were sorted using specific CD45, CD3 and CD8 staining. The dilution of CFSE on CD8+ T cells was determined by FCM.

### ScRNA-seq data acquisition

Single-cell RNA sequencing (scRNA-seq) raw data, comprising unique molecular identifier (UMI) matrix were downloaded from Gene Expression Omnibus (GEO) with accession number GSE169246. As previously described [23], 10 treatment-naïve patients diagnosed with advanced TNBC who received paclitaxel monotherapy based on clinical routine as first-line treatment. According to the Response Evaluation Criteria in Solid Tumors (RECIST), these patients were divided into responders (PR; P022, P011, P020, P008, and P013) and non-responders (SD/PD; P025, P018, P023, P024, and P003). Matched tumor biopsy and peripheral blood samples were collected before and after chemotherapy for scRNA-seq sequencing.

### Data reprocessing for dimension reduction and unsupervised clustering

R v4.0 (Seurat) was applied to analyze the raw UMI matrix through reserving high-quality cells with mitochondrial gene counts less than 20% and filtering out genes detected in less than five cells and cells with fewer than five genes. Next, based on the raw UMI counts, the normalization of matrix data following the normalization of total counts per cell was calculated, scaled by 1*e*6 and logarithmically transformed. The dispersion-based method, Seurat (FindVariableFeature) was used to calculate the highly variable genes (HVGs) and selected the top 2000 HVGs, further regressing out the unwanted variation, such as total counts and outliers of mitochondrial gene proportion from normalized matrix data. Principal component analysis (PCA) for dimension reduction was performed, and the top 20 primary components were selected for subsequent unsupervised clustering. Based on PCA, the Louvain algorithm was used to construct the SNN graph and determine clustering at a resolution of 0.6 for the first-round (re-clustering for individual cell types at a resolution of 0.4). Uniform Manifold Approximation and Projection (UMAP) or t-Distributed Stochastic Neighbor Embedding (t-SNE) projections were performed for cluster visualization.

### Identification for cell type

Cell-type markers were obtained from the CellMarker 2.0 database (http://bio-bigdata.hrbmu.edu.cn/CellMarker/) for identification. Each cluster was manually assigned cell types in terms of normalized gene expression using the following canonical markers: endothelial cells (PECAM1, CLDN5), T cells (CD3D, CD3E, CD3G, TRBC1), myeloid cells (LYZ, MNDA) and cancer cells (FAM177A1, B3GNT2, TRPS1). Further, we re-clustered endothelial cell and T cell types individually. Each EC subcluster was also defined by the top 100 markers (Additional file 2: Table S1), such as EC_subcluster2 (JUNB, IFITM1, HLA-C, HLA-DPA1) and EC_subcluster4 (PTPRC, MALAT1, ITGA4). Likewise, T cells were re-clustered at same resolution matched with ECs. T lineage subcluster identification was determined by the top 100 dominant markers, for example, exhausted T_subcluster0 (CD8, LAG3, NR4A2, and CXCL13) and Treg_subcluster1 (CD4, TNFRSF18, TNFRSF4, and GK).

### DEGs and functional enrichment analysis

In this study, the DEGs between pre- and post-treatment from responders/non-responders were screened by the Seurat (FindAllMarkers) function (|log2FC| > 0.5, adjusted P-value < 0.05). To compare the changes in the immune pathways of EC or T cell subtypes under different conditions, the enrichment score of genes involved in immune pathways was obtained using escape package (ssGSEA). For revealing biologic nature of cell subclusters, GSEA was performed by the clusterProfiler and similarly, Gene Ontology (GO) enrichment analysis was performed by clusterProfiler (bubble plot) and GOplot (circular plot) package.

### Correlations analysis

To reveal the correlation between different molecules, according to the average expression of genes in cell subclusters from the scRNA-seq dataset, Pearson’s correlation was performed using the R software. A customizable functional module for correlation analysis on the GEPIA2 website (http://gepia2.cancer-pku.cn) was investigated. To further elaborate on the relationship of TNFR2 with ECs or subtypes of ECs, according to the specific marker genes of cell populations, the score of each cell of ECs or EC_sub2 was calculated by escape package (ssGSEA). Through regression with a linear module between the total score of cell population and TNFR2 expression, P-value and co-efficient of determination R2 (R-squre) were obtained.

### Spatial transcriptomics data acquisition and spatial visualization

The raw unique molecular identifier (UMI) matrix of spatial transcriptomics (ST) data was obtained from GEO via the accessible number GSE210616, including 43 samples from 22 patients with primary TNBC [24]. A procedure similar to scRNA-seq data reprocessing, like normalization of gene expression counts, technical bias correction, further dimensionality reduction and clustering, and spatial visualization of either cell types or molecules at each image site was performed by R software according to positive definition for cell types or gene expression levels, respectively. The score of individual cell type were calculated by ssGSEA based on cell type markers from CellMarker 2.0. The portion of cells located in regions with a score greater than the top 25% were defined as positive expression; otherwise, it was defined as negative. Of note, any imaging site could concurrently show positivity for multiple cell types.

### Join count analysis

For the sake of quantifying spatial correlation between different cell types, join count analysis (JCA) developed by Bassiouni et al. [24] was introduced in this study and stJoincount (https://github.com/Nina-Song/stJoincount) was used for quantitation of spatial relationships between two types of cells. Briefly, a unique coordinate position for each image site in the samples was set using getcoord function of stJoincount. Spatial dependencies were quantified for each cell-type pair. We assigned a cell-type pair to the image site, even if the cell types were both scored in a shared site, by randomly assigning one cell type to image sites with the coordinate (x, y) value (x + y) being even, and another cell type to those with the coordinate (x, y) value (x + y) being odd (Additional file 1: Fig. S3F). The spatial distance between cell types was then determined quantitatively using the stJoincount package as previously described [24].

### Human protein atlas

Immunohistochemical images of TNFR2 proteins in human breast cancer tissues were obtained from the Human Protein Atlas (HPA) website (https://www.proteinatlas.org/ENSG00000028137-TNFRSF1B) to detect their distribution around the vessel regions.

### Protein–protein interaction networks

The database of STRING (https://cn.string-db.org/cgi/input.pl) was searched to explore the protein interactions of “TNFR2” with immune checkpoints involved in T cell-regulatory pathways through publicly integrated both experimental interaction results and computational interaction predictions. In the “Multiple Proteins” channel, TNFR2 and molecules involved in pathway “negative regulation of activated T cell proliferation” was inputted.

### Statistical analysis

All data were analyzed using GraphPad Prism software (RRID: SCR_002798). Bioinformatic plots and statistical analyses were carried out using R software (version 4.0; RRID: SCR_001905). Comparisons were performed using unpaired Student’s t-test, Two-way ANOVA, or Friedman test. Data were expressed as the mean ± SD. Statistical significance was set at P-value < 0.05.

## Results

### Identification of an immunoregulatory endothelial subcluster associated with PTX treatment failure

To characterize the polarization and transcriptomic dynamics of ECs exposed to PTX, scRNA-seq was performed on tumor/blood samples obtained from patients with TNBC pre-treatment (Pre) and biopsy samples 2 weeks post-treatment (Post) as previously described [23], including both responders (R) and non-responders (NR).

**Fig. 1:**
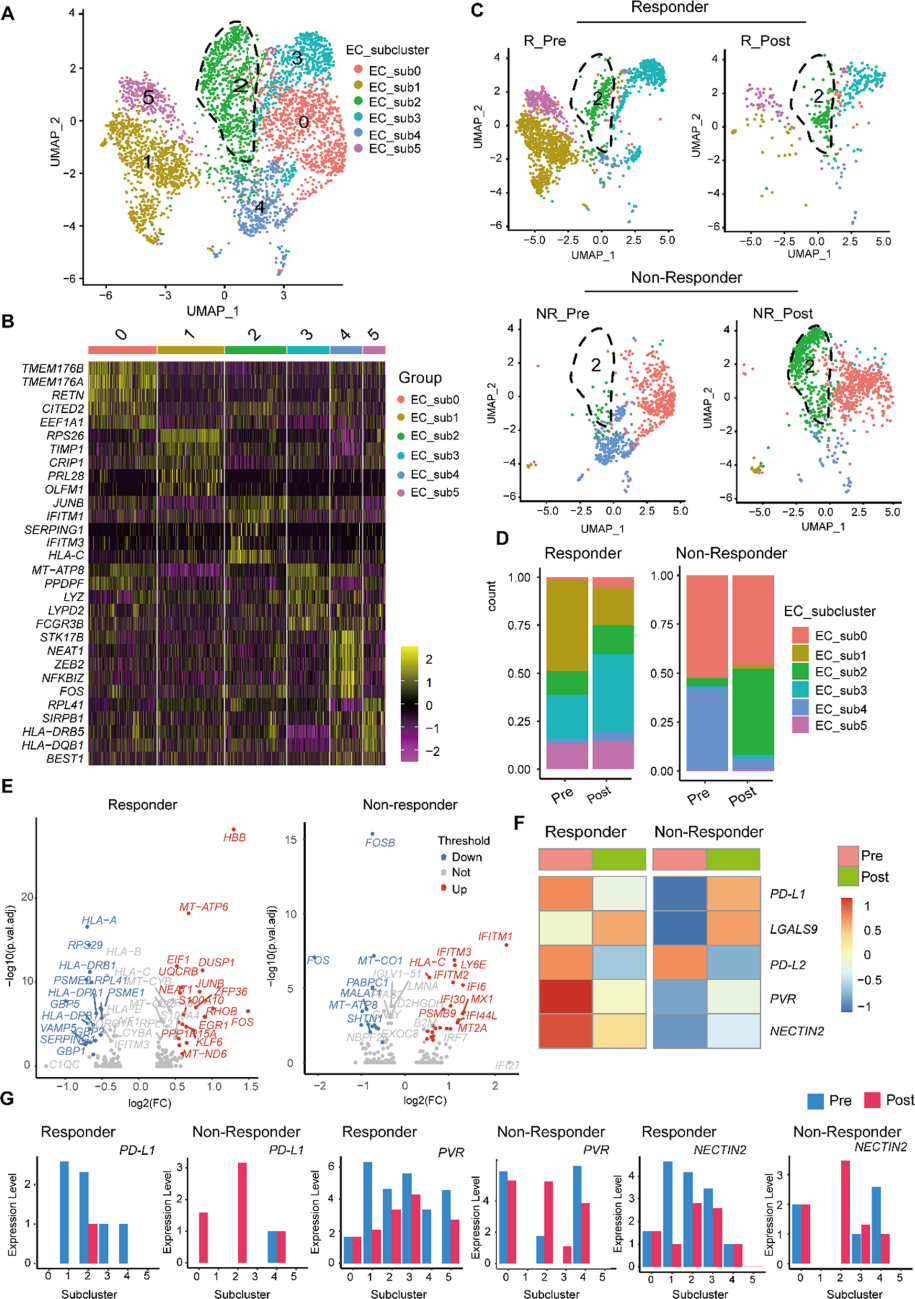
Identification of an immunoregulatory EC subtype participating in efficacy of PTX therapy. **A** UMAP plot of ECs, color-coded by subcluster. **B** Heatmap showing the expression levels of the top five marker genes belonging to each EC subcluster. Color scale: yellow, high expression; purple, low expression. **C** UMAP plots showing treatment-induced alterations in EC subclusters in responders (R) and non-responders (NR). **D** Proportions of each EC subcluster at different timepoints and in different therapeutic responses. **E** Volcano plots of EC_sub2 differential gene expression pre- versus post-treatment. Red, upregulated; dark blue, downregulated; gray, no significant change. The magnitude of differential expression (log2 FC) is shown on x-axis; the false discovery rate adjusted p-values are shown on the y-axis. **F** Heatmap of the expression patterns of representative immune checkpoints on the whole ECs. The color density reflects the average expression of a given gene. **G** Expression of inhibitory ligands according to EC subcluster

A t-distributed stochastic neighbor embedding (t-SNE) approach enabled the visualization of all cell clusters (Additional file 1: Fig. S1A) and cell distribution in the corresponding patients (Additional file 1: Fig. S1B). Heterogeneous clusters were identified using biomarkers and distinct gene signatures of T cells (CD3D, CD3E, and CD3G), ECs (platelet endothelial cell adhesion molecule 1 and claudin 5), and endothelial precursor cells (CD36) (Additional file 1: Fig. S1C and S1D). We set the modest resolution to 0.4 for ECs based on unsupervised clustering, and graph-based clustering analysis revealed six subclusters, EC_subcluster 0–5, each possessing distinct gene signatures (Fig. 1A, B; Additional file 2: Table S1). The increased proportion of EC subcluster2 (EC_sub2) after PTX therapy was associated with non-responders (Additional file 1: Fig. S2A). As shown in Fig. 1C, D, EC_sub2 significantly increased in size following PTX therapy in non-responsive patients, implying a possible role for this subcluster in negatively correlating with PTX therapy efficacy.

To analyze each EC subcluster in drug-resistant settings, GO enrichment of the top 100 DEGs was performed to identify the top 25 specific GO terms enriched in each subcluster. For EC_sub2, four GO terms were involved in inflammation response and immune regulation, including antigen processing and presentation and response to IFNγ, suggesting an involvement of IFNγ-related signaling in modulating endothelial immunogenicity (Additional file 1: Fig. S2B). This was also supported by the higher expression of the major histocompatibility complex (MHC) class I/II genes *HLA-C* and *HLA-DPA1* in EC_sub2 from non-responsive tumors (Additional file 1: Fig. S2C). Further, EC_sub2 was characterized by the marker gene HLA-C (Fig. 1B), which showed the highest level of expression among subclusters (Additional file 1: Fig. S2D) and indicate the immunomodulation of this subtype on T cells.

Transcriptome profiling of responders and non-responders identified numerous transcriptionally regulated genes and revealed that the MHC genes *HLA-A*, *HLA-DPB1*, and *HLA-DRB1* decreased in responders, but HLA-C increased in non-responders (Fig. 1E), similar to Fig. S2C. In addition, gene regulators, such as *IFITM3*, *PSMB9* and IFITM2*,* were associated with the immunogenic nature of EC_sub2 in non-responders (Fig. 1E; Additional file 3: Table S2). As semi-professional APCs, ECs modulate immune responses mainly through cell surface molecules [6, 25], such as PD-L1/2, poliovirus receptor (PVR), nectin cell adhesion molecule 2 (NECTIN2) and galectin-9 (encoded by *LGALS9*). Analysis of whole ECs showed that the expression of inhibitory molecules *PD-L1*, *PVR*, and *NECTIN2* decreased in responders and increased in non-responders following treatment (Fig. 1F). Furthermore, EC_sub2 cells from non-responders preferentially expressed genes encoding the ligands *PD-L1*, *PVR*, and *NECTIN2* following PTX treatment (Fig. 1G). The inverse pattern was observed in responsive tumors (Fig. 1G). Collectively, our findings warrant further exploration of the functional mechanisms underlying immunoregulation of T cells by EC_sub2.

### EC-mediated suppression of CD8+ T cells via PD-1/PD-L1 inhibitory axis

**Fig. 2:**
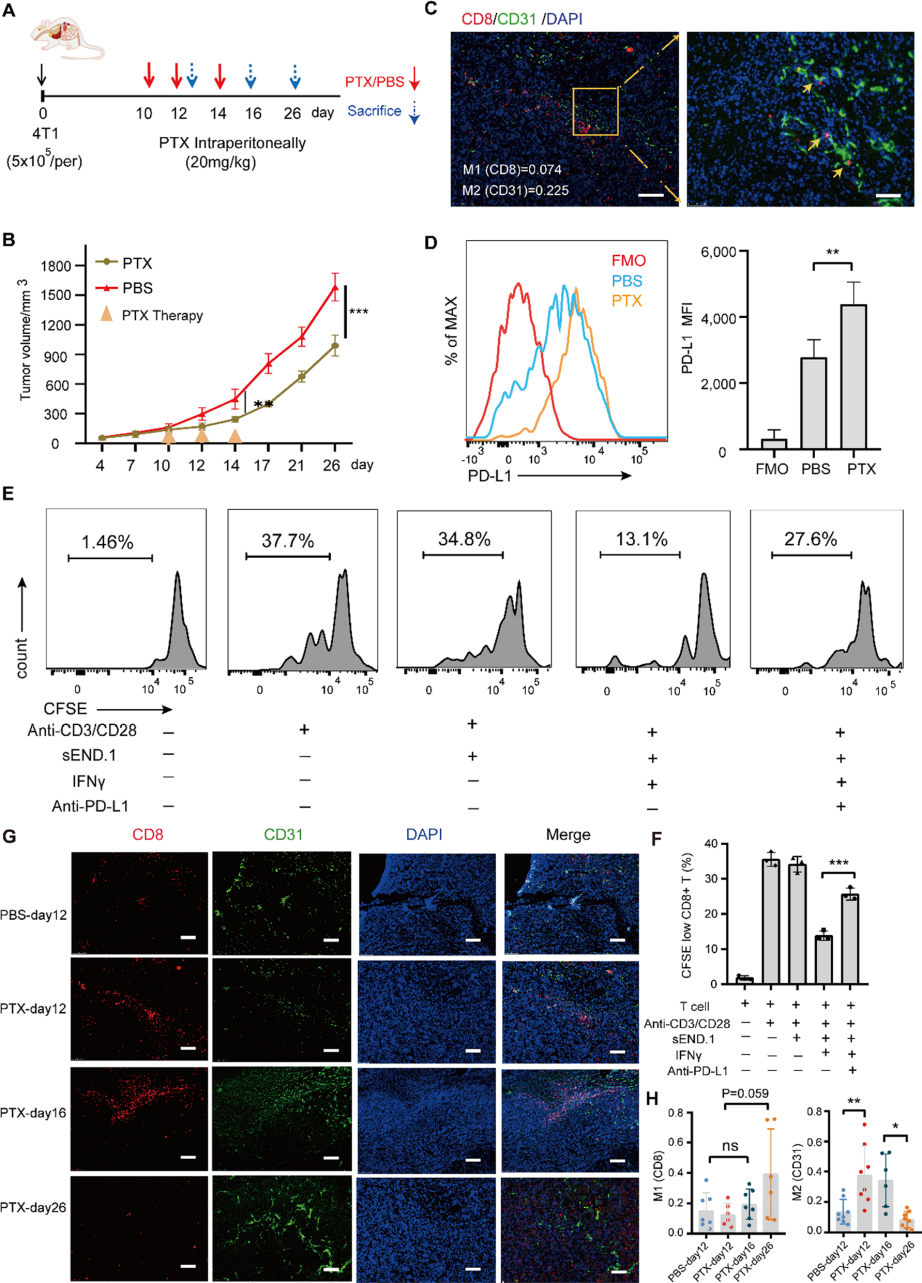
Endothelial PD-L1 mediates suppression of CD8+ T cell within tumor endothelium. **A** Orthotopic 4T1-bearing murine model. **B** Tumor growth curves in both groups; the yellow arrows indicate PTX or PBS administration. **C** Representative images of tumor samples (day 26). Immunofluorescence staining of CD31 (green), CD8 (red), and DAPI (blue); yellow arrows indicate cell–cell interactions between CD8+ T cells (CD8) and ECs (CD31). Scale bars: 1 mm (left) and 200 µm (right). **D** Representative histograms and quantification of PD-L1 expression on ECs from 4T1-bearing mice (day 26). Fluorescence Minus One (FMO) served as a negative control. **E** The splenocytes from OT-I mice were incubated with IFNγ-stimulated sEND.1 at a ratio of 500:1 with or without pre-administration of anti-PD-L1 antibody (10 μg/ml) 1 h prior to co-incubation. 72 h later, the proliferation of CD8+ T cells was detected by flow cytometry using the FITC channel. **F** The quantitation of CD8+ T cell prefoliation. n = 3; **G** immunofluorescence staining of CD31 (green) and CD8 (red) in tumor samples. The number of samples: PBS-day12, n = 7; PTX-day 12, n = 6; PTX-day 16, n = 7; PTX-day 26, n = 6. Scale bars: 200 µm. **H** Values of M1 (CD8) and M2 (CD31) over time corresponding to **G**. *P < 0.05, **P < 0.01, ***P < 0.001; ns, not significant

The mechanism by which tumor-related ECs regulate T cell activity upon chemoresistance remains unclear. Spatial limitation is a crucial consideration for the interaction of infiltrating T cells with surrounding APCs in the TME, particularly regarding the function of ECs on T cells. An orthotopic 4T1-transplanted murine model was established to investigate spatial considerations, and the mice were treated with PTX or PBS as a control (Fig. 2A). PTX inhibited tumor growth over the 26-day experimental period (Fig. 2B). Using immunofluorescence staining of CD31 and CD8 in tumor samples isolated from PTX-treated mice, Fig. 2C shows the close contact (yellow arrow) between ECs and CD8+ T cells, suggesting that spatial contact may be a footstone for immunomodulation of CD8+ T cells by ECs.

To further depict the detailed and quantitative spatial tendency of endothelial cells toward CD8+ T cells in TNBC, a spatial transcriptomic dataset from patients diagnosed as advanced TNBC [24] was obtained from GEO (see “Methods and materials”). High-resolution (0.6) unsupervised clustering revealed a more comprehensive histological and genetics-based annotation within a single specimen. For example, the graph-based clustering of sample 092A revealed ten clusters (Additional file 1: Fig. S3A and S3B) and the corresponding genetic signatures for each cluster (Additional file 1: Fig. S3C). ESTIMATE analysis revealed that cluster_02 had the highest scores for both T cells and ECs, followed by clusters _03 and _07 (Additional file 1: Fig. S3D). Spatial visualization confirmed their highly contiguous localization (Additional file 1: Fig. S3E). Furthermore, to better quantify the spatial exclusion and dependency between different cell types, a novel algorithm called join count analysis (JCA) was used to delineate the directionality and degree of spatial proximity between several cell-type pairs, including T cells and ECs. The results showed that T cells presented high spatial proximity to ECs, macrophages, and B cells, but were distant from cancer cells and fibroblasts (Additional file 1: Fig. S3F and S3G). The high spatial proximity of T cells to ECs in TME implies their potential close interactions, and T cell functions might be regulated by ECs.

CD8+ T cell-dependent immune responses are the core component of PTX-triggered antitumor effects [26, 27]. Next, we analyzed T cell characteristics in patients receiving PTX therapy. Eight T cell subclusters (Additional file 1: Fig. S4A; Additional file 4: Table S3), four involved CD8+ T cells (exhausted_subcluster0, effector_subcluster2, central memory_subcluster3, and naïve_subcluster5), three involved CD4+ T cells (Treg_subcluster1, central memory_subcluster4, and naïve_subcluster6), and one was undefined (Cytotoxic T-KLRB1_sucluster1) were identified. The proportion of cells in CD8_subcluster0 increased post-treatment in tumors that were not responsive to chemotherapy and decreased in responsive tumors (Additional file 1: Fig. S4B and S4C), placing CD8+ exhausted T cells as the key lynchpin in determining the PTX efficacy.

T cell exhaustion involves multiple inhibitory receptors, such as programmed cell death protein (PD)-1, lymphocyte-activation gene (LAG)3, and T cell immunoreceptor with Ig and ITIM domains (TIGIT). The expression of *PD-1* and *LAG3* in subcluster_0 from non-responders increased following treatment, whereas their expression decreased following treatment in responders (Additional file 1: Fig. S4D). Although *IFNγ* has long been considered critical for CD8+ T cell activity, its expression decreased in responders and increased in non-responders (Additional file 1: Fig. S4D). *PD-1* showed the strongest correlation with *TIGIT* (r = 0.50), followed by *LAG3* (r = 0.38) (Additional file 1: Fig. S5E). ssGSEA identified the “PD-1 signaling” pathway as being increased in responders and decreased in non-responders (Additional file 1: Fig. S4F). These results suggest a strong positive relationship between the emergence of exhausted CD8+ T cells and resistance to PTX therapy, which may be mediated by inhibitory PD-1 signaling.

Considering the high spatial proximity of ECs to CD8+ T cells (Additional file 1: Fig. S3), enhanced IFNγ signaling in EC_su2 from non-responders (Additional file 1: Fig. S2B) and increase of IFNγ expression by exhausted CD8+ T cells (Additional file 1: Fig. S4D), the susceptibility of EC-mediated immunoregulation to IFNγ was investigated. GSEA identified the “IFNγ-mediated signaling pathway” as being increased in responders but decreased in non-responders (Additional file 1: Fig. S5A and S5B). Though remaining almost unchanged in responder post-treatment, the expression of *IFNγ* was primarily within T, natural killer, and cancer cell populations in non-responders (Additional file 1: Fig. S5C).

Negative feedback has been considered to enable adaptive resistance to targeted cancer therapy through regulating the inflammatory activity [28]. We have previously demonstrated that anti-tumor CD8+ T cell responses depend on IFNγ-triggering destruction of blood vessels [11]. The upregulation of endothelial PD-L1 and IDO-1 mediated by IFNγ is a key feedback mechanism to avoid uncontrolled T cell responses [29, 30]. In this study, the level of PD-L1 expression on tumor-related ECs derived from PTX-treated mice is higher than those isolated from mice that did not receive PTX than those isolated from mice that did not receive PTX (Fig. 2D). In vitro, PD-L1 expression on ECs could be induced by IFNγ in a dose-dependent manner (Additional file 1: Fig. S5D). CD8+ T cell proliferation assays were carried out after co-incubation of IFNγ-stimulated sEND.1 cells with spleen cells isolated from OT-I mice at a ratio of 1:500. Consistent with previous findings [17, 31, 32], CD8+ T cell proliferation was inhibited by IFNγ-stimulated ECs; an anti-PD-L1 antibody partially alleviated this inhibition (Fig. 2E, F). This suggests that IFNγ-mediated immunosuppression by EC is partly via PD-L1.

Since ECs can inhibit CD8+ T cells through the inhibitory PD-L1/PD-1 axis, we investigated whether spatial exclusion of CD8+ T cells within the endothelial region occurred during PTX therapy. In this study, using Manders co-localization coefficients (MCC) analysis (see “Methods and materials”), fluorescence staining of CD8 (referred as M1) and CD31 (referred as M2) in 4T1 cell-transplanted tumors was performed to explore the space overlap. As shown in Fig. 2C, G, CD8 (red) and CD31 (green) were stained by immunofluorescence and overlapped in some niches (M1 = 0.074 and M2 = 0.255), indicating that 7.4% of CD8+ T cells co-localized with CD31+ ECs, and 25.5% of CD31+ ECs were in close contact with CD8+ T cells, implying that interactions could occur. Analysis of murine tumor tissues harvested 12, 16, and 26 days after tumor cell injection (2, 6, and 16 days after the initiation of PTX treatment, respectively), showed an initial increase, followed by a gradual reduction in CD8+ T cell homing to the tumors (Fig. 2G). To quantify spatial overlap during chemotherapy, MCC analysis revealed an initial sharp rise, followed by a gradual decrease in M2 (Fig. 2H), suggesting that the ratio of endothelium containing CD8+ T cell infiltration to all endothelium initially increased and subsequently decreased.

Initiation of PTX may initially boost CD8+  T cell migration from the peripheral blood to tumors; however, with the progressive expression of endothelial PD-L1, interactions with ECs may subsequently mediate contact exclusion, causing a reduction in the number of CD8+ T cells distributed within the endothelium. The IFNγ-mediated PD-L1 expression by ECs was suitable for CD8+ T cell inhibition, making it a possibility for tumor cells to drive immune escape.

### TNF–TNFR2 signaling strengthens PD-L1-dependent endothelial immunosuppression

Disturbances in the tumor inflammatory microenvironment may lead to chemoresistance. TNFα is one of the major inflammatory factors in TME and whether TNFα and its receptors are involved in chemoresistance is unclear. It has been documented that, although TNFα alone did not greatly influence PD-L1 expression by ECs, it can further promote PD-L1 expression in the context of IFNγ [17]. Given the increased interferon gamma-mediated signaling in EC_sub2 from non-responders (Fig. S2B), this program reminds us that TNFα in the TME, may contribute to T cell suppression via endothelial immune-regulatory mechanisms.

**Fig. 3:**
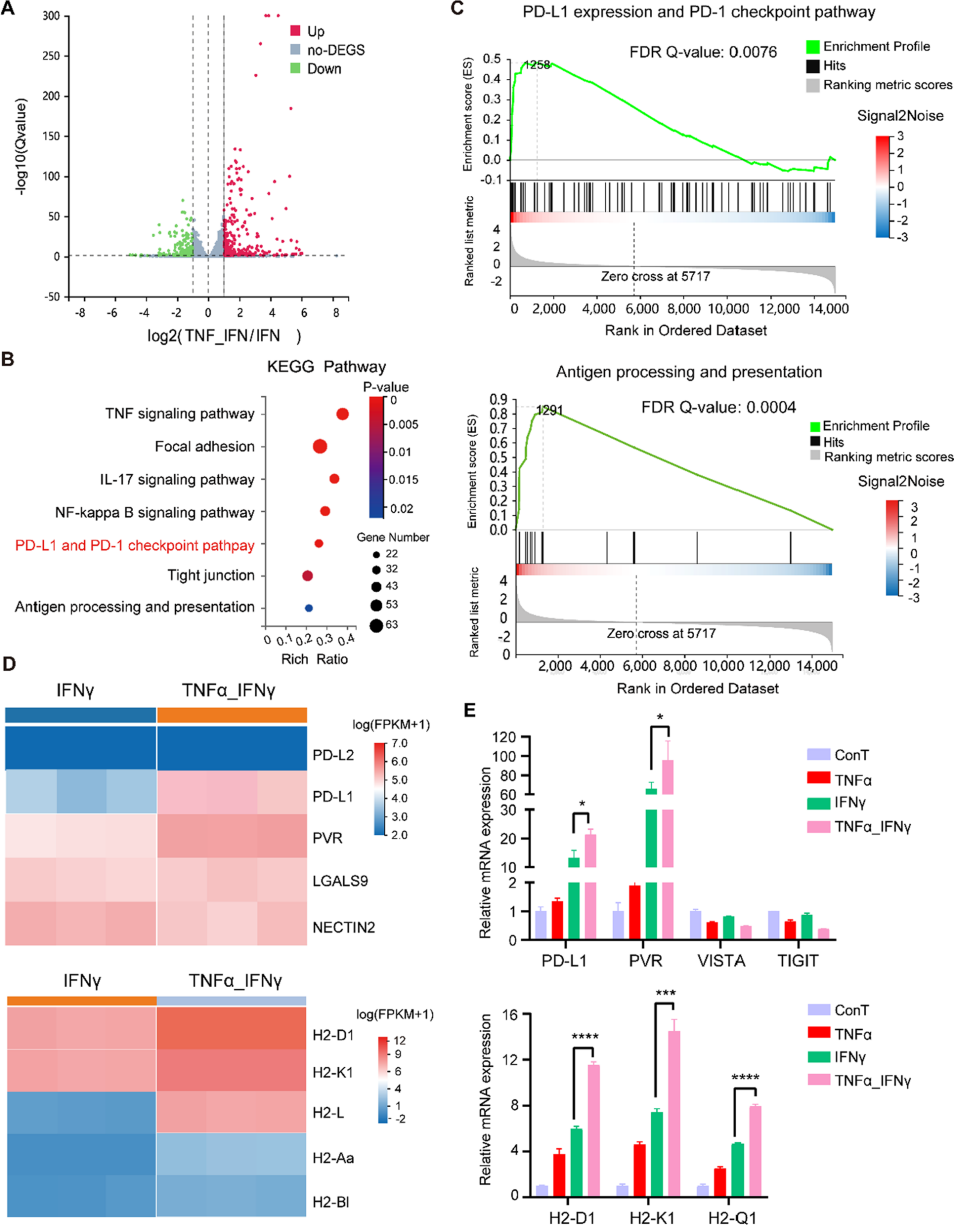
TNFα promotes PD-L1-dependent immunosuppressive property of ECs. **A** Volcano map revealing differentially expressed genes (DEGs) from RNA-seq dataset of IFNγ-stimulated sEND.1 cells treated with or without TNFα. **B** Significantly enriched pathways according to KEGG analysis of genes from the RNA-seq dataset. **C** Distinct expressions of immune checkpoints and MHC molecules. **D** GSEA terms linked to “PD-L1 expression and PD-1 pathway” and “antigen processing and presentation” pathway. **E** Increased transcription of immune checkpoints and MHC-associated molecules in sEND.1 cells mediated by TNFα; n = 3. *P < 0.05, ***P < 0.001, ****P < 0.0001

In order to explore the immune characteristics of ECs possibly driven by TNFα, firstly, RNA-sequencing (RNA-seq) of sEND.1 cells was conducted after administration of IFNγ and TNFα or IFNγ alone as control. Transcriptomic profiling identified numerous genes regulated by TNFα including 2978 upregulated and 3389 downregulated genes (Fig. 3A). KEGG pathway analysis revealed enrichment of PD-L1 expression and PD-1 checkpoint pathway (Fig. 3B). The expression of *Pd-l1* were markedly elevated (Fig. 3C). GSEA of ECs treated with TNFα and IFNγ revealed a striking enrichment of the “PD-L1 expression and PD-1 checkpoint pathway” (FDR Q value of 0.0076) and “antigen processing and presentation” (FDR Q value of 0.0004) (Fig. 3D). qRT-PCR revealed that TNFα could cause a significant increase in *Pd-l1* transcription of ECs when combined with IFNγ (Fig. 3E).

**Fig. 4:**
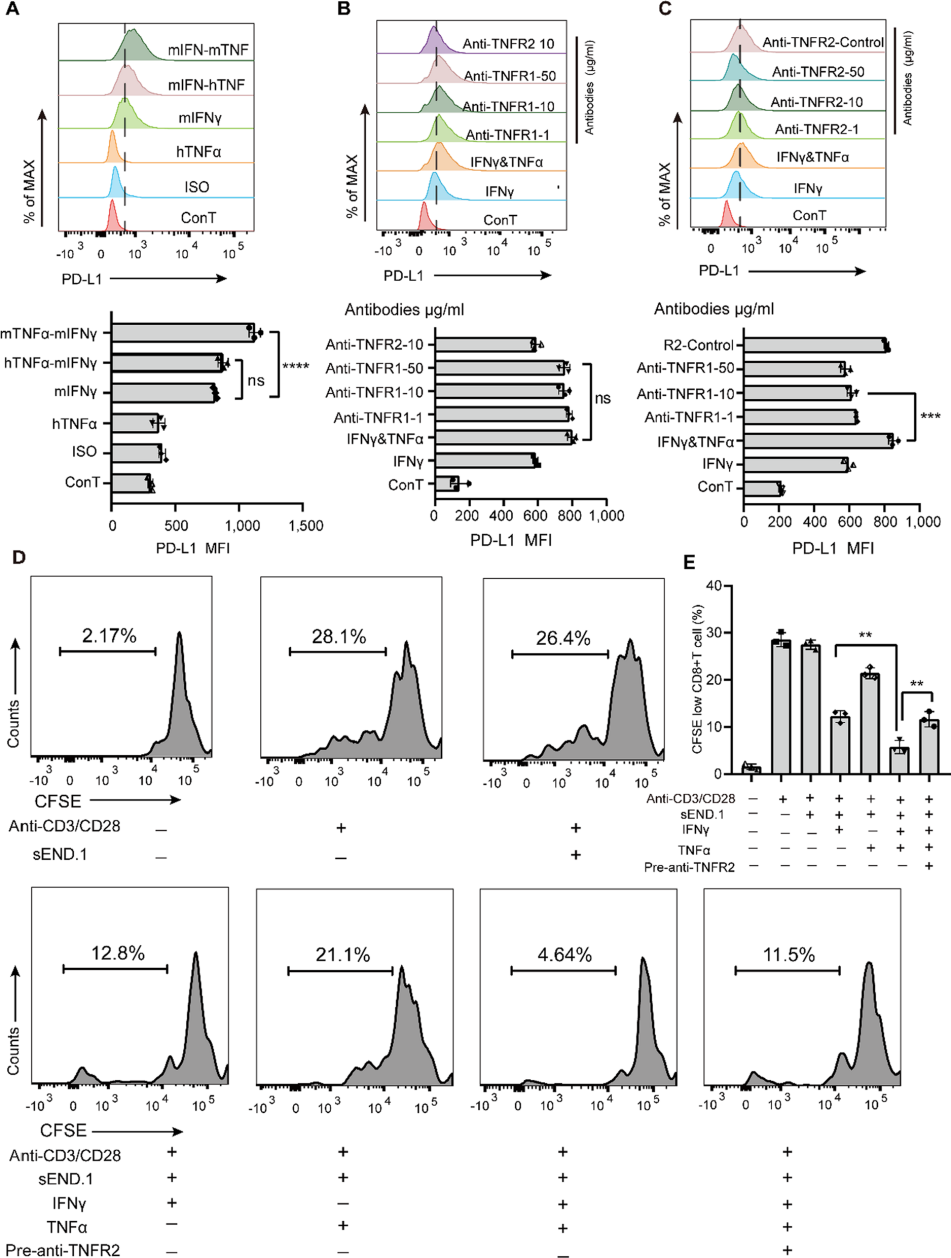
Endothelial immunosuppression is enhanced by TNFR2 but not TNFR1. **A** Representative histograms and quantification of PD-L1 expression by sEND.1 cells following administration of either human or mouse TNFα with mouse IFNγ; n = 3. **B** Representative histograms and quantification of PD-L1 expression by ECs incubated with IFNγ, TNFα and anti-TNFR2; n = 3. **C** Representative histograms and quantification of PD-L1 expression by sEND.1 cells incubated with IFNγ, TNFα, and anti-TNFR1; n = 3. **D** Proliferation of OT-I CD8+ T cells detected by FCM using the FITC channel after incubation with IFNγ-stimulated sEND.1 cells treated by TNFα with or without anti-TNFR2. **E** Quantification of CD8+ T cell proliferation; n = 3. **P < 0.01, ***P < 0.001, ****P < 0.0001; ns, not significant

To investigate which receptor of TNFα determines endothelial PD-L1-dependent immunosuppression, a series of in vitro experiments were performed. Reportedly, human TNFα (hTNFα) can bind TNFR1, but not TNFR2, on murine ECs [33]. We compared the effects of hTNFα and mouse TNFα (mTNFα) on PD-L1 expression to determine the contribution of TNFR2. PD-L1 expression of ECs was not altered by hTNFα but was increased by mTNFα (Fig. 4A). Moreover, blocking the TNF–TNFR2 interaction with an antibody abolished the effect of mTNFα on PD-L1 expression (Fig. 4B), whereas blocking the TNF–TNFR1 interaction had no similar effect (Fig. 4C).

Therefore, the in-depth relationship between TNFR2 and immunosuppression of ECs warrants further exploration. Pairwise Spearman’s correlation coefficients demonstrated TNFR2 was more strongly correlated with EC_sub2 than with the total EC population (Additional file 1: Fig. S6A).

GO analysis showed enrichment of EC_sub2 marker genes in T cell-related pathways, indicating that TNFR2 was negatively involved in the “T cell activation” pathway (Additional file 1: Fig. S6B). In EC_sub2 cells, the expression of *LGALS9*, *HVEM*, and *PD-L1* was positively and closely associated with *TNFR2* (Additional file 1: Fig. S6C). Analysis of the GEPIA2 dataset confirmed a strong positive correlation between *TNFR2* and several ligands, such as HLA-C (Additional file 1: Fig. S6D). TNFR2 and other molecules involved in the GO term “negative regulation of activated T cell proliferation” were entered into the STRING database, revealing a protein–protein interaction network with TNFR2 at its center surrounded by inhibitory molecules including PD-L1 and LGALS9 (Additional file 1: Fig. S6E). Recently, a subtype of TNFR2-expressing endothelial precursor cell (EPC) with immunosuppressive property was identified [34, 35]. To further investigate the role of endothelial TNFR2 in CD8+ T cell suppression, ECs were incubated with CD8+ T cells. TNFR2 blockade significantly relieved the TNFα-enhanced inhibition of CD8+ T cells, partially restoring their proliferative capacity (Fig. 4D, E). Additionally, TNFα-stimulated ECs could inhibit CD8+ T cell proliferation to a certain extent, implying a direct role for TNFα, separate to effects on IFNγ-stimulated cells.

As shown in Fig. S7A, *TNF* signaling in EC_sub2 was enhanced following PTX treatment. However, the expression of genes involved in the TNFR2-NF-κB signaling pathway increased in EC_sub2 from non-responders but decreased from responders (Additional file 1: Fig. S7A). Interestingly, increased TNFα expression in cancer cells post-treatment appeared to be beneficial to PTX therapy (Additional file 1: Fig. S7B). In the murine model, although PTX reduced tumor growth to a certain extent, it also increased the proportion of TNFR2+ ECs on day 26, compared to the PBS control (Additional file 1: Fig. S7C and S7D). In addition, the UMAP plots showed a substantial expansion of TNFR2-expressing EC_sub2 cells post-treatment in non-responders (Additional file 1: Fig. S7E). Flow cytometry analysis revealed that PTX stimulation also increased the expression of TNFR2 in HUVEC and sEND.1 cells in vitro (Additional file 1: Fig. S7F and S7G).

These results indicate that the larger number of TNFR2-expressing ECs from non-responders might be attributed to PTX stimulation. Endothelial TNF–TNFR2 signaling may function as a bridge by which tumors facilitate the PD-L1-dependent immunosuppression of ECs to render PTX therapy ineffective.

### TNFR2 promotes PD-L1 expression through tuning glycolytic activity by activating NFκB signaling

**Fig. 5:**
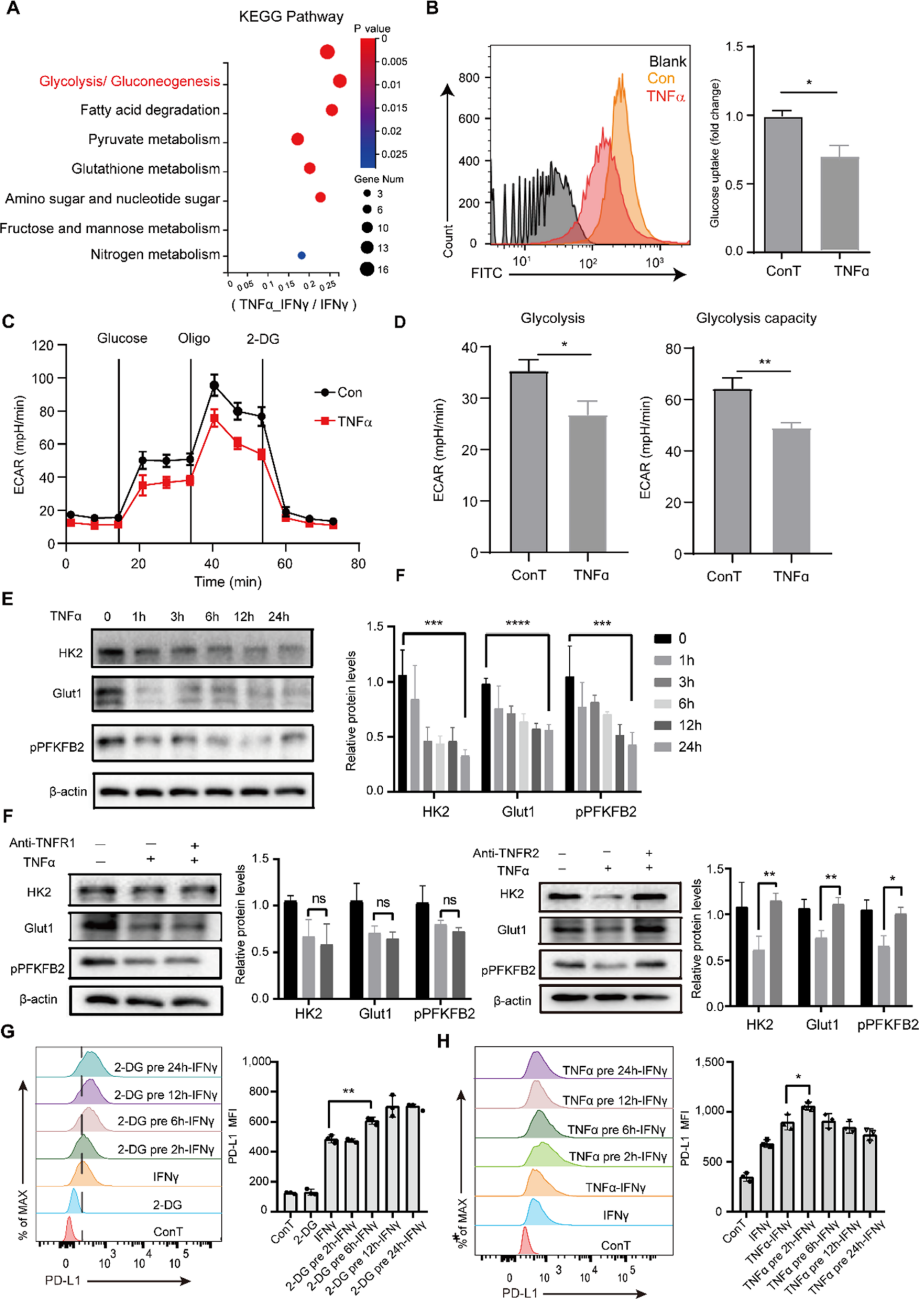
Tuning endothelial glycolysis by TNF–TNFR2 signaling facilitates PD-L1 expression. **A** Significantly enriched pathways according to KEGG analysis of genes from the RNA-seq dataset. **B** Representative histograms and quantification of glucose uptake by sEND.1 cells incubated with or without TNFα. **C** Time course and **D** quantification of glycolytic function in sEND.1 treated with or without TNFα. **E** Western blots showing glycolysis-associated proteins expressed by sEND.1 cells incubated with or without TNFα. n = 3. **F** Western blots showing proteins expressed by sEND.1 treated with or without TNFα and anti-TNFR1 (left) or anti-TNFR2 (right). n = 3. **G** Representative histograms and quantification of PD-L1 expression by sEND.1 treated with 2-DG following IFNγ stimulation. n = 3. **H** Representative histograms and quantification of PD-L1 expression by sEND.1 cells treated with TNFα prior to IFNγ stimulation; n = 3. *P < 0.05, **P < 0.01, ***P < 0.001, ****P < 0.0001

An increasing number of studies have focused on cellular immunometabolism-related PD-L1 upregulation [20, 36]. To further understand the metabolic mechanisms through which the TNF–TNFR2 interaction contributes to endothelial immunosuppression, RNA-seq analysis on sEND.1 cells was performed. KEGG analysis of the RNA-seq dataset revealed significant enrichment of pathways related to energy metabolism, such as “glycolysis and gluconeogenesis” and “fatty acid degradation” (Fig. 5A), implying that TNFα may regulate PD-L1 expression through the glycolytic pathway. To experimentally verify these findings, we investigated the impact of TNFα on glycolytic uptake in ECs. Clearly, glucose uptake was decreased in TNF-treated ECs (Fig. 5B), as was glycolytic metabolism (Fig. 5C, D) and the expression of glycolysis-associated proteins (Fig. 5E). Next, we found that TNFR2 blockade significantly restored glycolysis by upregulating the expression of HK2, Glut1, and phosphorylated PFBFK2 (pPFKFB2) proteins (Fig. 5F).

**Fig. 6:**
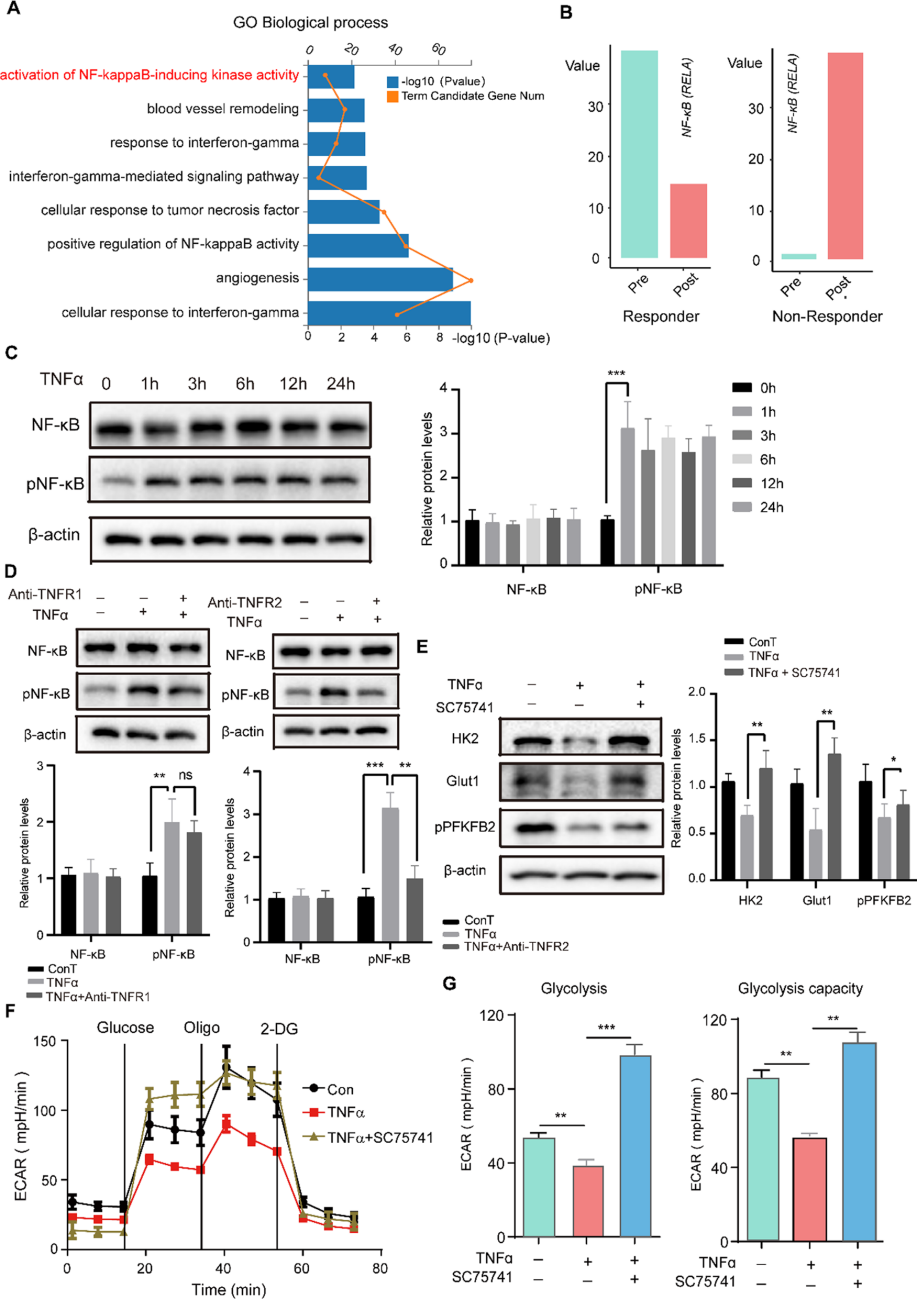
TNFR2-decreased endothelial glycolysis is NF-κB-dependent. **A** Significantly enriched inflammatory pathways by GO analysis from the RNA-seq dataset. **B** Bar graphs showing expression of NF-κB (RELA) in EC_sub2. **C** Expression and phosphorylation state of NF-κB in sEND.1 cells treated with or without TNFα assessed by WB. n = 3. **D** Expression and phosphorylation state of NF-κB in sEND.1 cells treated with or without TNFα and anti-TNFR1 (left) or anti-TNFR2 (right). n = 3. **E** Expression of glycolysis-associated proteins by sEND.1 cells treated with or without TNFα and NF-κB (RELA) inhibitor (SC75741) measured by WB. n = 3. **F**, **G** Time course (**F**) and quantification (**G**) of glycolytic function of sEND.1 detected by the Seahorse Analyzer; n = 3. *P < 0.05, **P < 0.01, ***P < 0.001

To further understand the cellular mechanisms by which TNFR2 alters glycolytic activity in ECs, RNA-seq analysis was conducted. The NF-κB signaling pathway was the most significantly enriched term according to GO analysis (Fig. 6A). NF-κB has been shown to regulate TNF-induced expression of PD-L1 in various cell types [37, 38]. NF-κB (RELA) expression decreased in EC_sub2 from responders and increased in EC_sub2 from non-responders (Fig. 6B). TNFα treatment increased phosphorylation of NF-κB (p65) (Fig. 6C), which was partially reversed by anti-TNFR2 (Fig. 6D). Inhibiting NF-κB abolished TNFα-inhibited glycolytic metabolism (Fig. 6E–G). These data suggested that NF-κB may represent a key component of the TNF–TNFR2 signaling pathway influencing glucose metabolism.

How TNF-reprogrammed glucose metabolism involves the process of IFNγ-induced PD-L1 expression needed further investigated. PD-L1 expression was analyzed in terms of glucose metabolism. Although 2-deoxy-d-glucose (2-DG), a non-metabolizable glucose analogue, had no obvious effect on PD-L1 expression by ECs, pre-inhibition of glycolysis by 2-DG further enhanced PD-L1 expression by ECs treated with IFNγ (Fig. 5G). Similarly, pre-treatment with TNFα to curb glucose uptake and metabolism enhanced PD-L1 expression by ECs (Fig. 5H). Thus, tuning glycolysis of ECs by TNFα is prepared for higher upregulation of PD-L1 expression subject to IFNγ treatment.

### TNFR2-expressing ECs are spatially proximal to exhausted CD8+ T cells

**Fig. 7:**
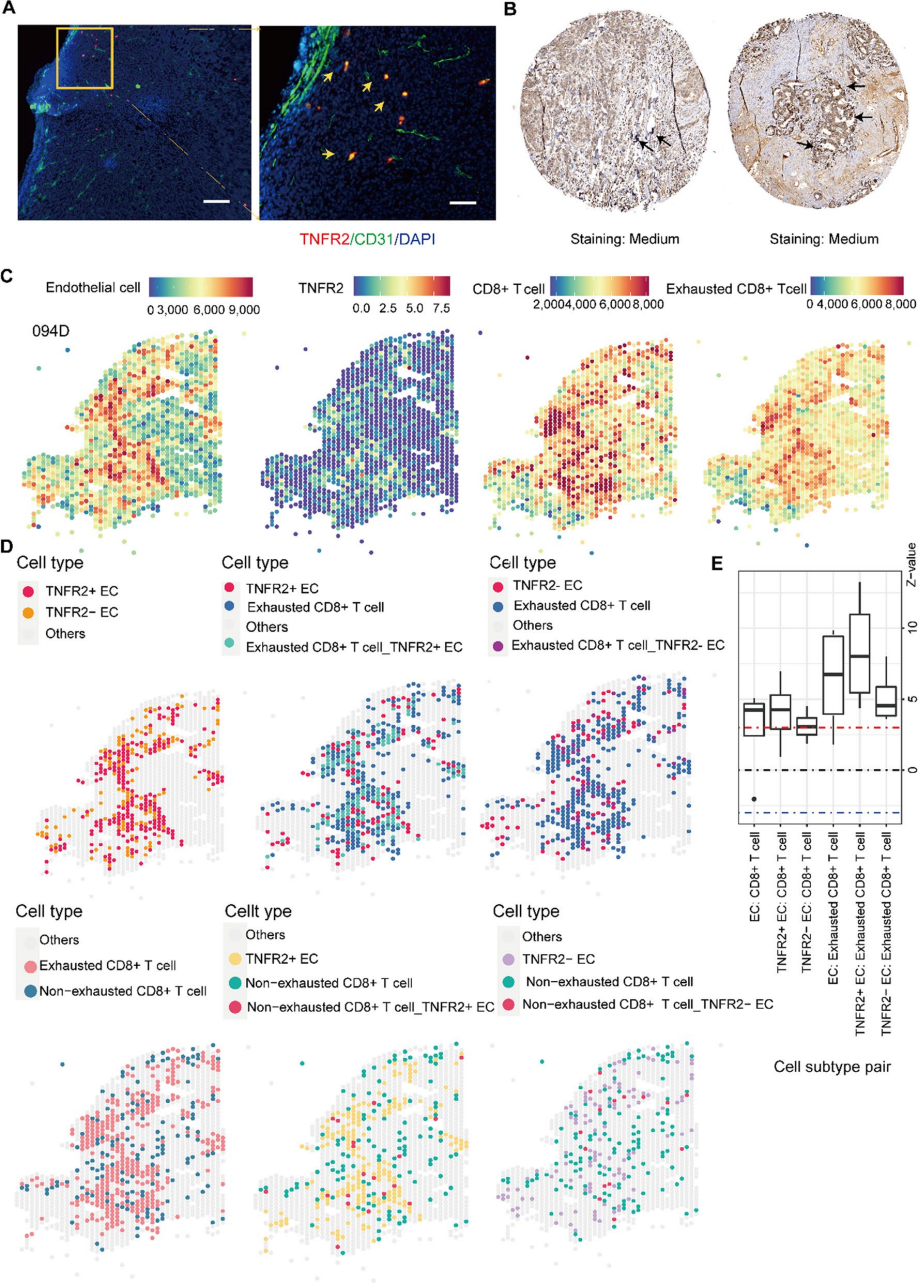
TNFR2 + ECs present spatial proximity to exhausted CD8+ T cells. **A** Images of immunofluorescence staining for TNFR2 (red) and CD31 (green); Yellow arrows indicate TNFR2 and CD31 co-localization. Scale bar: 1 mm (left) and 200 µm (right). **B** Representative immunohistochemistry images of TNFR2 distribution in breast cancer tissues from HPA; Black arrows indicate TNFR2 expression on blood vessels. **C** Spatial visualization of ECs, *TNFR2*, CD8+ T cells and exhausted CD8+ T cells in the representative sample 094D. **D** Spatial visualization of TNFR2+ and TNFR2− ECs, exhausted and non-exhausted CD8+ T cells, and their overlapping regions. Colored by cell type assignment. **E** A summary of the resulting z-scores of cell subtype pairs represented in a box plot calculated by JCA. Vertical line represents two extreme points of the z-values in a wide spectrum; a vertical line in the box marks the mean; the box shows the interquartile range of the z-value. Z > 0, positive dependency, indicating spatial aggregation; Z < 0, negative dependency, indicating spatial dispersion. Z value reflects the degree of spatial deviation of the observed join count between cell pairings; when it is close to 0, z value indicates no spatial dependency, which is a state of near-random distribution. The z-value presenting significance of the results was also selected at cutoff of + 3 visualized between the dotted lines

Next, we investigated whether the effect of TNFR2+ ECs on T cell exhaustion was reflected in their spatial relationship. The distribution of TNFR2 in TNBC tissues was investigated. Staining for CD31 and TNFR2 in the vascular compartments of tumor-bearing mice following PTX therapy showed considerable co-localization of TNFR2 and ECs (Fig. 7A). Analysis of the Human Protein Atlas (HPA) revealed that TNFR2 was intensively distributed in vascular regions (Fig. 7B).

The spatial distribution of TNFR2+, TNFR2− ECs and exhausted, non-exhausted CD8+ T cells was then visualized (Fig. 7C, D). The exhausted CD8+ T cell/TNFR2+ EC overlapping region (cyan) was greater and more intense than that (purple) of exhausted CD8+ T cells/TNFR2− ECs (Fig. 7D). No obvious overlapping regions were observed between TNFR2+ ECs and non-exhausted CD8+ T cells (Fig. 7D). JCA revealed that although both TNFR2+ and TNFR2− ECs were spatially correlated with exhausted CD8+ T cells, the former showed a more significant positive spatial relationship with exhausted CD8+ T cells than the latter (Fig. 7E).

We validated the high spatial localization of TNFR2+ ECs and exhausted CD8+ T cells, providing a pathological basis for the rational combination of PTX agent with TNFR2 blockade.

### Blocking TNFR2 signaling improves the efficacy of PTX therapy

Previously, TNFR2 blockade has been reported to enhance anti-tumor immunity in combination with anti-PD-L1 therapy in a breast cancer model [39]. As PTX therapy may be affected by TNF–TNFR2 signaling, we investigated the effects of TNFR2 blockade on PTX efficacy.

**Fig. 8:**
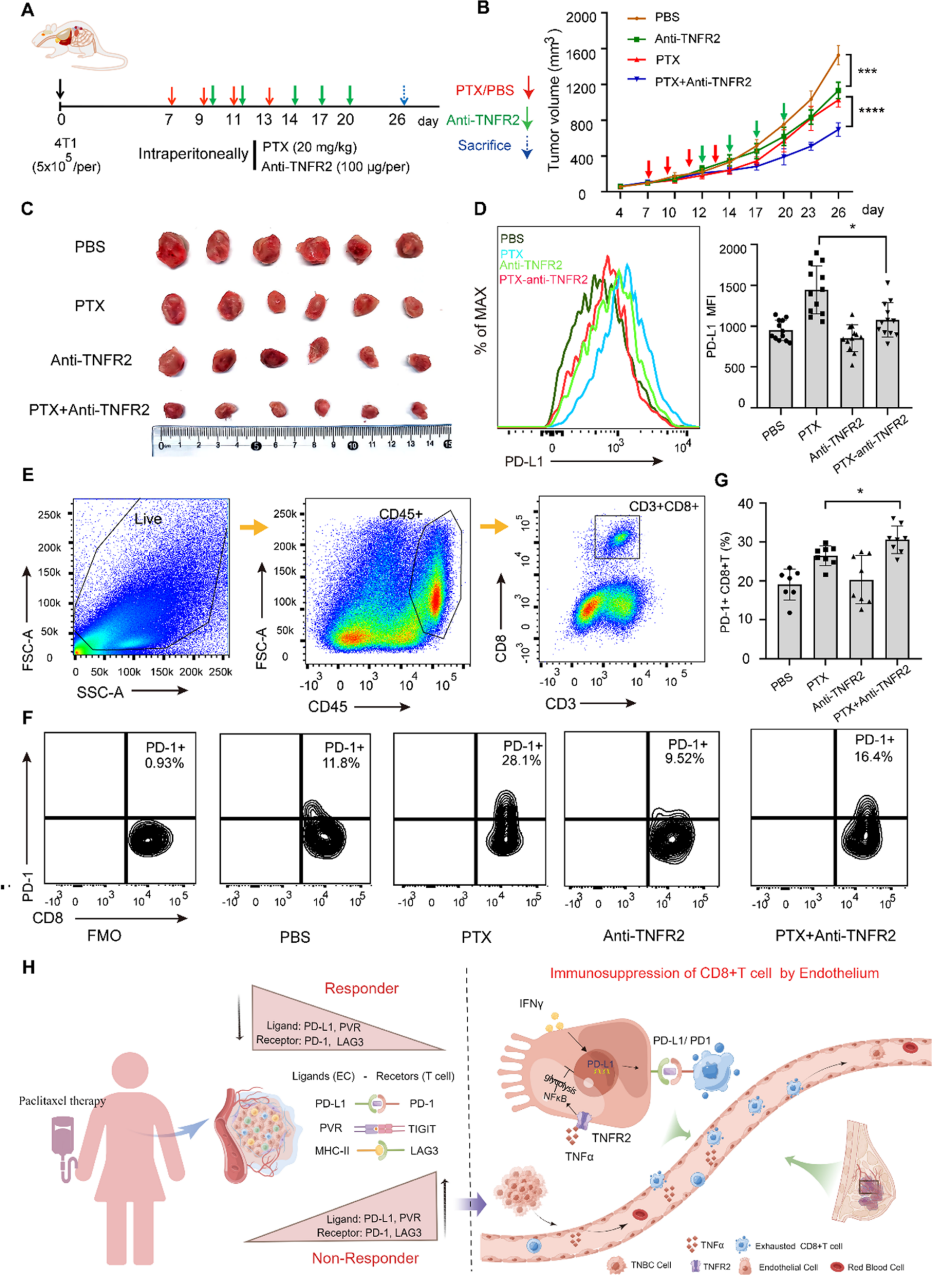
TNFR2 blockade and PTX combination therapy. **A** Schematic representation of 4T1-transplanted murine model and therapeutic procedure. **B** Tumor growth curves. **C** Primary tumors harvested from mice. **D** Representative histogram (left) and quantification (right) of PD-L1 expression on tumor ECs (CD45−CD31+PD-L1+) isolated from mice. **E**, **F** Gating scheme for (**E**) CD45+CD3+CD8+ T cells and (**F**) PD-1+ T cells (CD45+CD3+CD8+PD-1+) from tumor-bearing mice. **G** Quantification of the proportion of PD-1+CD8+ T cells. **H** Schematic illustration showing immune features of CD8+ T cells and ECs from responders and non-responders following PTX therapy. The expression of the ligands PD-L1, PVR, and MHC class II molecules on ECs and the corresponding receptors PD-1 and LAG3 on CD8+ T cells increase in non-responders but decrease in responders. Endothelial TNF–TNFR2 signaling enables inhibition of CD8+ T cell through PD-L1/PD-1 axis via regulating cellular NF-κB–glycolysis pathway on the basis of IFNγ-mediated immune feedback. Schematic illustration was created on the website of “Home for Researchers”. *P < 0.05, ***P < 0.001, ****P < 0.0001

A 4T1-transplanted murine model was used to test the efficacy of the anti-TNFR2 monoclonal antibody (mAb) and PTX combination therapy (Fig. 8A). The TNFR2 mAb significantly improved the efficacy of PTX, inhibiting the growth of primary tumors and prolonging the survival of tumor-bearing mice (Fig. 8B, C) compared to controls. To gain a more comprehensive understanding of immune alterations within the TME, tumor-infiltrating lymphocytes and intratumoral ECs were isolated from these mice, which revealed a decreased proportion of CD45−CD31+PD-L1+ immunosuppressive ECs (Fig. 8D and Additional file 1: Fig. S8A) and increased the infiltration of CD8+ T cells into the TME confirmed by IHC and FCM technologies (Additional file 1: Fig. S8B–D) in mice treated with combination therapy. As indicated previously, PD-1 serves as a biomarker of T cells exhaustion [40]. PD-1 expression was reduced on CD8+ T cells from the tumors of mice treated with combination therapy (Fig. 8E–G), suggesting that the exhaustion of CD8+ T cells associated with PTX therapy failure was alleviated to some extent. Collectively, these findings demonstrate that both decreased PD-L1 expression by ECs and PD-1 expression by CD8+ T cells may represent an improvement in the immunomodulatory state within the endothelial compartment following combination therapy.

## Discussion

Although PTX can trigger a variety of CD8+ T cell-dependent anti-tumor immune responses [26, 27], it is not suitable for the combination treatment with atezolizumab (an anti-PD-L1 antibody) [41, 42]. The mechanism of non-responsiveness to combination regimes remains incompletely inventoried. These PD-1-concomitant exhaustion-relevant genes (e.g. TIGIT and LAG3) may partially explain the incompatibility. Our scRNA-seq analysis revealed that in addition to the PD-1/PD-L1 pathway, other inhibitory cell surface molecules were also expressed by exhausted CD8+ T cells and ECs in non-responsive tumors, indicating the existence of a PD-1/PD-L1-independent inhibitory axis. Except for PD-L1, other checkpoints PD-L2, Galectin9 and HVEM [43] have been reported in ECs. In this study, the upregulation of T cell exhaustion-related genes in non-responders, including *PD1*, *TIM3*, and *LAG3*, following chemotherapy may be primarily due to persistent stimulation by tumoral antigens derived from the ICD program [2, 40]. Although PTX has been recommended in combination with pembrolizumab (anti-PD1) for the clinical treatment of TNBC [44], further research on the effects of PTX on inhibitory cell surface molecules is required.

The heterogeneity of ECs enables them to perform various functions in the TME. Our results on the diversity of EC subtypes may explain, at least in part, the contradictory findings of previous studies which have shown ECs suppress tumor growth through drug infusion [45] or enhance tumor growth through endothelial immunosuppression [12]. In this study, the TNFR2+ ECs subtype induced exhaustion and inhibited CD8+ T cells proliferation via PD-L1. In fact, the antitumor immune response decreased with the continuation of chemotherapy, as shown by the reduced presence of CD8+ T cells within the tumor endothelium. This was partly due to the prevention of T cell extravasation into the tumor parenchyma by TNFR2+ ECs, suggesting that the endothelium acted as an immunological, not just a physical, barrier.

We propose that endothelial immunoregulation and its spatial relationship with T cells may concertedly belong to two facets of a linked vascular activity. Compared with their functional properties, the spatial features of ECs are often ignored. We analyzed the spatial dynamics underlying the process of EC-mediated suppression and exhaustion of CD8+ T cells during resistance to PTX therapy; TNFR2 seemed able to determine this program. TNFR2+ ECs showed greater spatial proximity to exhausted CD8+ T cells, supporting the notion that the function of a molecule depends on its spatial properties. Spatial redistribution of CD8+ T cells around blood vessels was observed throughout PTX administration, providing a new spatial perspective for evaluating the efficacy of chemotherapy. Although single-cell spatial transcriptomes have been applied to unmask various features of cells across many cancer types, the spatial dynamics of the TME in therapeutic settings remain poorly studied.

Although glycolysis is linked to various biological processes of endothelial cells, the relationship between glucose metabolism and EC immunogenicity remains unclear. Substantial evidence has highlighted the role of glycolysis in the regulation of angiogenesis [46], and the suppression of T cells [19]. This study showed that pre-inhibition endothelial glycolysis by TNF further boosts IFNγ-induced PD-L1 expression, perhaps because cellular state of glycolysis tuning sensitized ECs, resulting in more glycolysis substrates which positively correlates with PD-L1 expression. However, a few questions remain unanswered. For instance, lactate, a product of glycolysis, is required for PD-L1 expression by macrophages [20]. Whether pre-treatment with TNFα promotes lactate accumulation in ECs, thus upregulating PD-L1 expression, requires further investigation.

## Conclusions

In this study, we systematically analyzed the heterogeneity and immune features of EC subclusters in both responders and non-responders. Remodeling of the TME in non-responders involves both an increase in exhausted CD8+ T cells featured by upregulated expression of PD-1 and LAG3, and an immunoregulatory EC subtype characterized by enhanced TNFR2 expression. Endothelial TNFR2 inhibited CD8+ T cell proliferation by promoting a PD-L1-dependent feedback mechanism via activating NF-κB signaling and regulating glycolysis (Fig. 8H). This study highlights the potential value of targeting TNFR2 to overcome the immunological barrier of the endothelium for CD8+ T cells infiltration, increase tumor responsiveness to PTX therapy and facilitate its combination with immunotherapy.

### Supplementary Information

Supplementary Material 1.

Supplementary Material 2.

## Data Availability

The RNA-seq dataset on endothelial cells supporting the conclusions of this article is available at the China National Center for Bioinformation (CNCB) under accession ID CRA014876 (https://ngdc.cncb.ac.cn/gsa/browse/CRA014876). Additional data not presented in this study are available upon request.

## Abbreviations

TNBC: Triple-negative breast cancer
ECs: Endothelial cells
PTX: Paclitaxel
R: Responder
NR: Non-responder
Pre: Pre-treatment
Post: Post-treatment
TNFR2: Tumor necrosis factor receptor 2
PD-L1: Programmed death-ligand 1
PD-1: Programmed cell death 1
MCC: Manders’ colocalization coefficients
TNBC: Triple-negative breast cancer
GSEA: Gene set enrichment analysis
ssGSEA: Single-sample gene set enrichment analysis
RNA-seq: RNA-sequencing
ScRNA-seq: Single-cell RNA sequencing
t-SNE: T-distributed Stochastic Neighbor Embedding
UMAP: Uniform Manifold Approximation and Projection
HPA: Human Protein Atlas
GEO: Gene Expression Omnibus
KEGG: Kyoto Encyclopedia of Genes and Genomes
