# Supplementary material for: Tumor necrosis factor receptor 2 promotes endothelial cell-mediated suppression of CD8+ T cells through tuning glycolysis in chemoresistance of breast cancer
Source: J Transl Med. 2024 Jul 20;22:672. doi: 10.1186/s12967-024-05472-5 (PMC11265105; doi:10.1186/s12967-024-05472-5)
Supplement: Supplementary file 1 — Supplementary Material 1. [file 12967_2024_5472_MOESM1_ESM.docx]

**Supplementary Figure Legends**


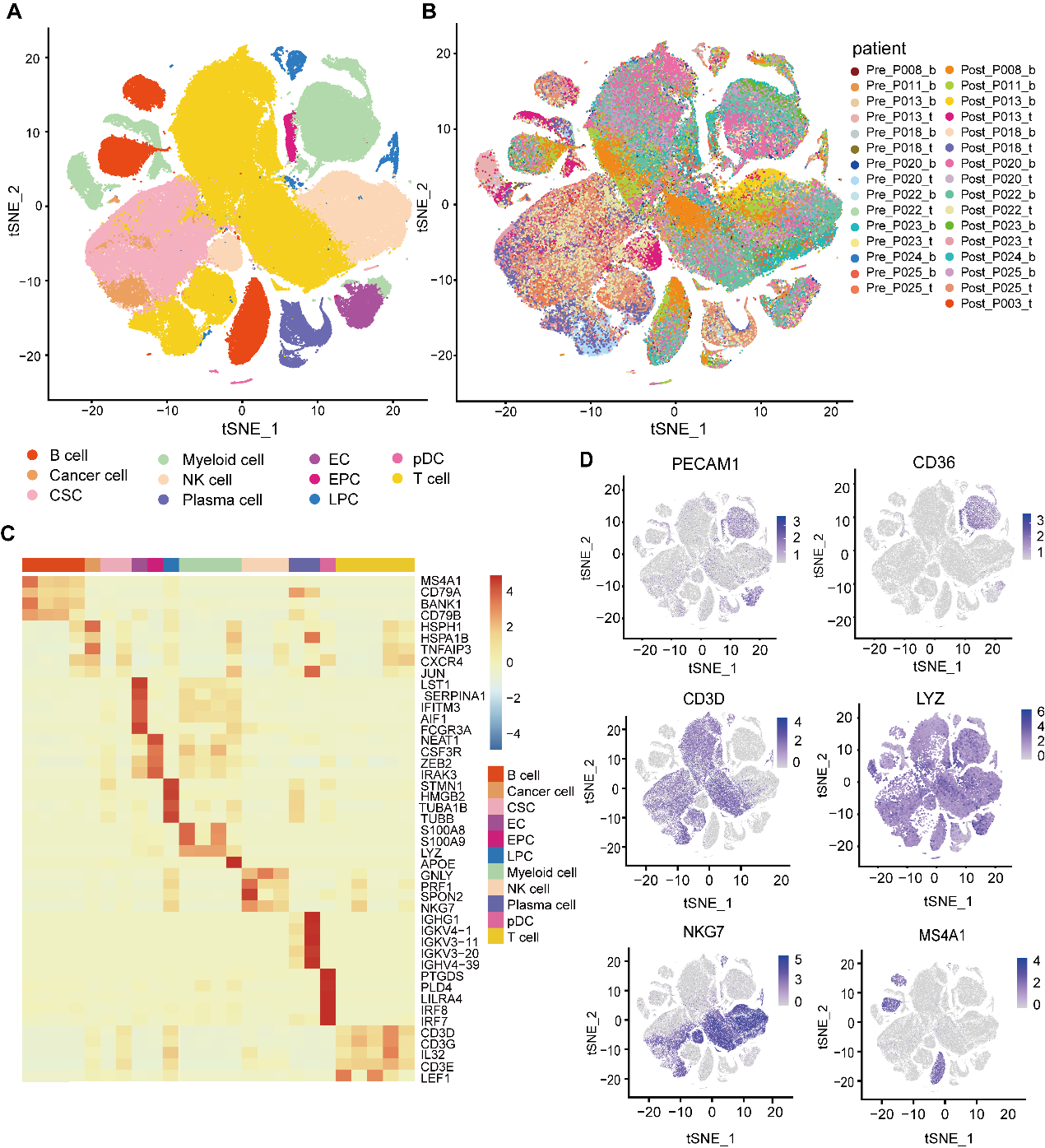


**Fig. S1** Single-cell landscape in TNBC responding to paclitaxel. **A-B,** t-SNE visualization of a total of 187,963 cells, color-coded by their cell type annotation (**A**) and origin of patients **(B**). “t” indicates tumor; “b” indicates blood. **C,** Gene-expression heatmap of signature genes in distinct cell clusters. **D,** t-SNE plot of markers for representative cell clusters, such as EC (PECAM1), EPC (CD36), T cell (CD3D), macrophage (LYZ), B cell (Ms4A1) and NK cells (NKG7).

Abbreviation: CSC, cancer stem cell; EPC, Endothelial precursor cells; LPC, Luminal progenitor cell; NK, Natural killer; pDC, Plasmacytoid dendritic cell.


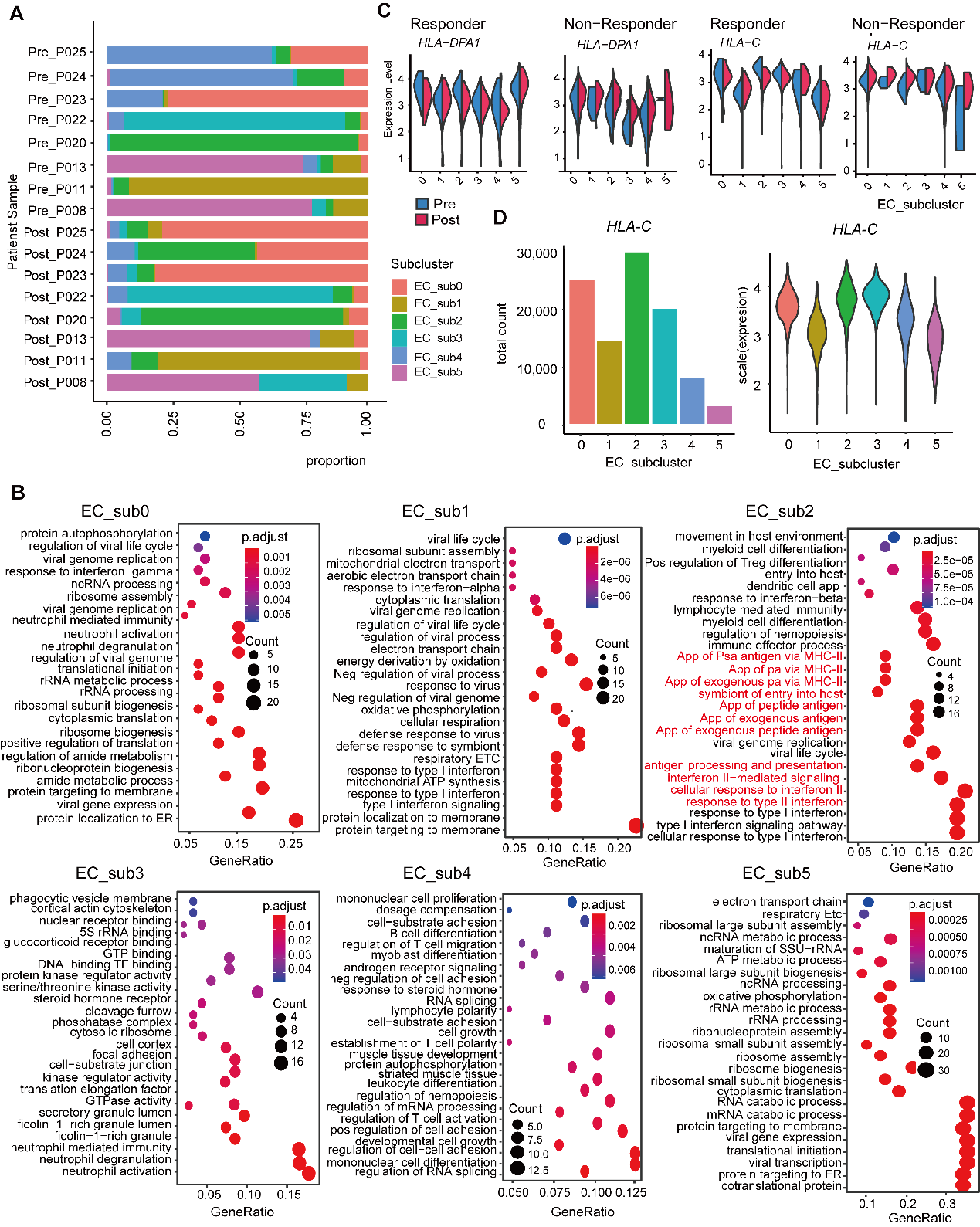


**Fig. S2** Transcriptomic and immunologic features of EC subclusters. **A,** Proportion of six EC subclusters cross differently treated patients (y-axis). **B,** The top-25 upregulated pathways per EC subcluster shown in Dot plots, was performed by GO enrichment analysis with a limitation of a p-value adjust < 0.01. Color scale indicates adjusted p-value; dot size represents the number of enriched genes within each GO term. **C,** Violin plots of the expression levels of the representative MHC-I/II molecules (*HLA-DPA1* and *HLA-C*) in each EC subclusters from responder or non-responder. **D,** Bar plot (left) and violin plot (right) showing the expression levels of HLC-C across six EC subclusters.

Abbreviations: App, antigen processing and presentation; ER, endoplasmic reticulum; Etc, electron transport chain; Neg, negative; Pos, positive; Pa, peptide antigen; Psa, Polysaccharide.


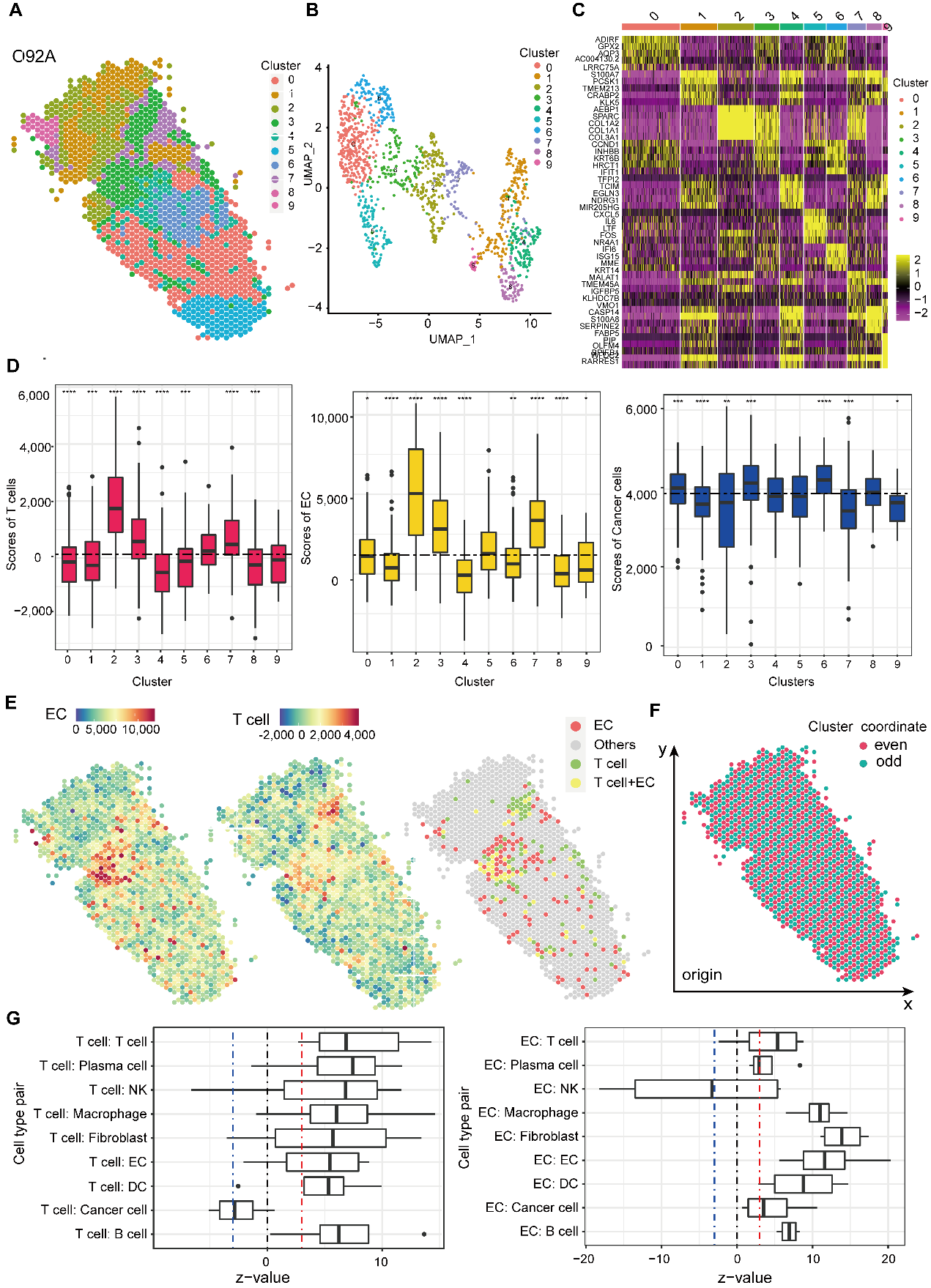


**Fig. S3** High spatial closeness of ECs to T cells. **A,** Spatial visualization of ten distinct clusters of samples 092A. **B,** Corresponding to (**A**), UAMP plot of ten clusters. **C,** Heatmap showing the expression levels of the top five marker genes across clusters. Color scale: yellow, high expression; purple, low expression. **D,** ESTIMATE scores of T, EC and cancer cell type in each subregion. The x-axis represents ten subregions and the y-axis represents ESTIMATE score of cell types. **E,** Graph-visualized EC (left) and T cell (middle) types and their co-localization (right, yellow) determined by ssGSEA. **F,** Spatial visualization of a cell-type pair coordinate (x, y) with the value (x+y) being even (red) and odd (green). **G,** Box plot of a summary of z values stemming from JCA to quantify the spatial relationship between distinct cell-type pairs.


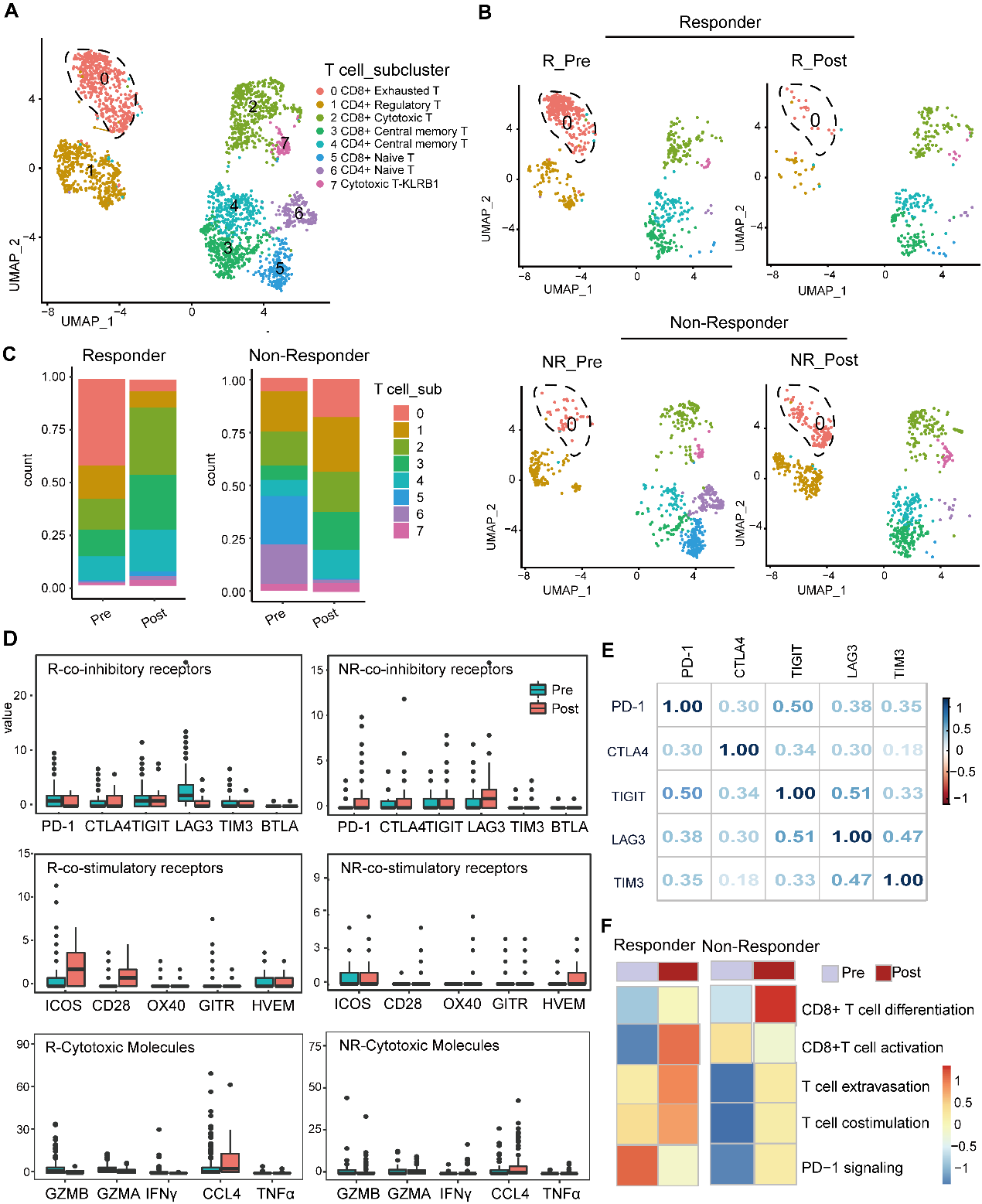


**Fig. S4** Clustering and features of T cell subclusters in TNBC treated with PTX. **A,** The UMAP projection of single T cells, showing eight T cell subclusters. Each dot corresponds to a single cell, colored according to cell cluster. **B,** UMAP plots revealing the comparison of each subcluster pre- and post-chemotherapy in R or NR, color coded for T cell subclusters. **C,** Proportions of each T cell subcluster at different timepoints and in different therapeutic responses. **D,** Pre- and post-treatment expression of co-inhibitory receptors, co-stimulatory receptors, and cytotoxic molecules in CD8_subcluster0. **E,** Pearson correlation analysis of multiple immune checkpoints expressed by CD8_subcluster0 from non-responders. **F,** Heatmap of immune-related pathways enriched in CD8_subcluster0, as identified by ssGSEA. Color scale represents enrichment scores: red, high expression; blue, low expression.


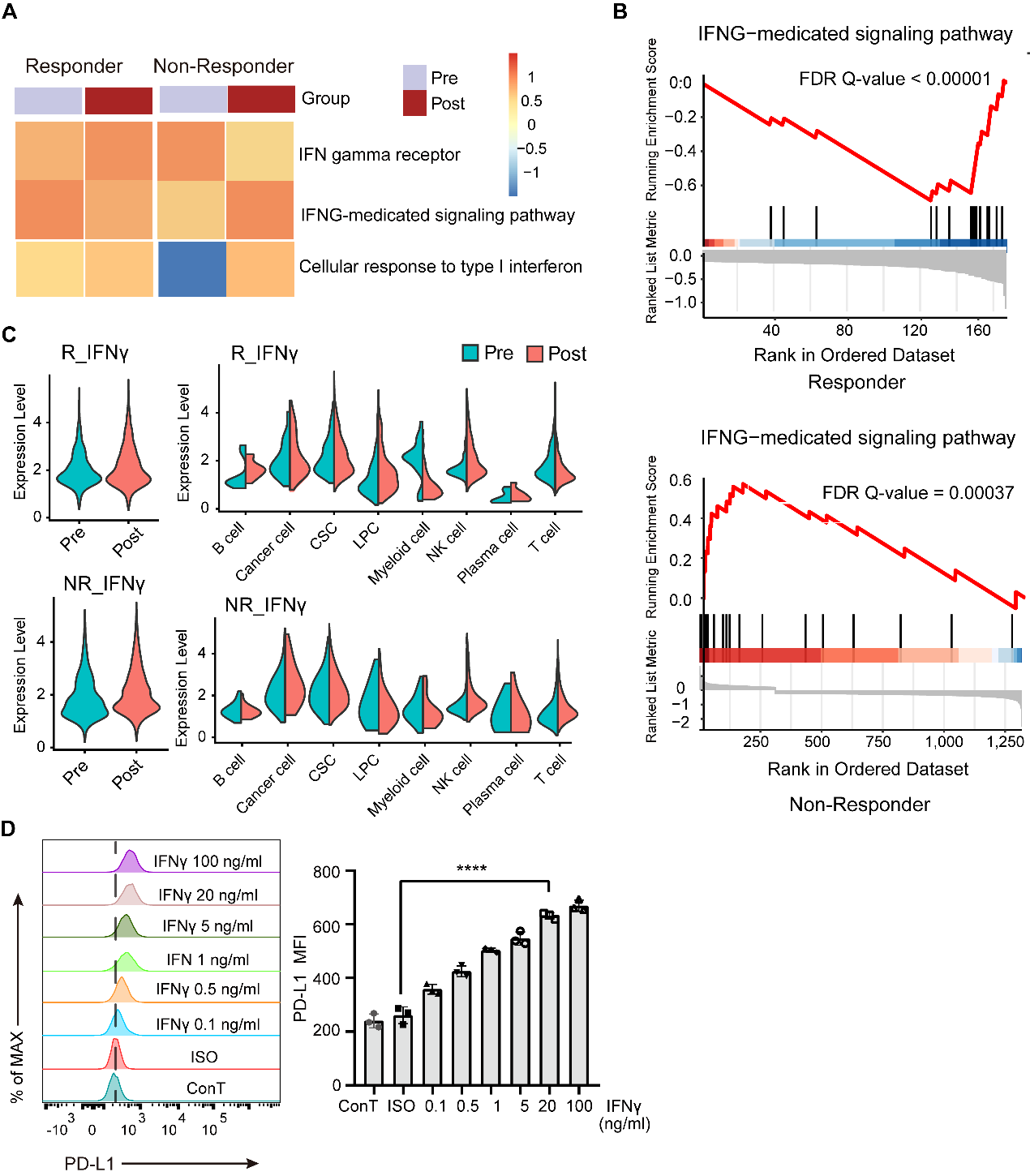


**Fig. S5** IFNγ-induced endothelial PD-L1 exerts immunosuppressive activity. **A,** Heatmap of IFNγ-related signaling pathways enriched in EC_sub2. **B,** GSEA plot of “IFNG-mediated signaling pathway” in EC_sub2 from responder or non-responder. **C,** Expression levels of *IFNγ* in the whole (left) and each cell types (right). **D,** PD-L1 expression by sEND.1 stimulated by IFNγ at a range of concentrations; n = 3. ****P < 0.0001.


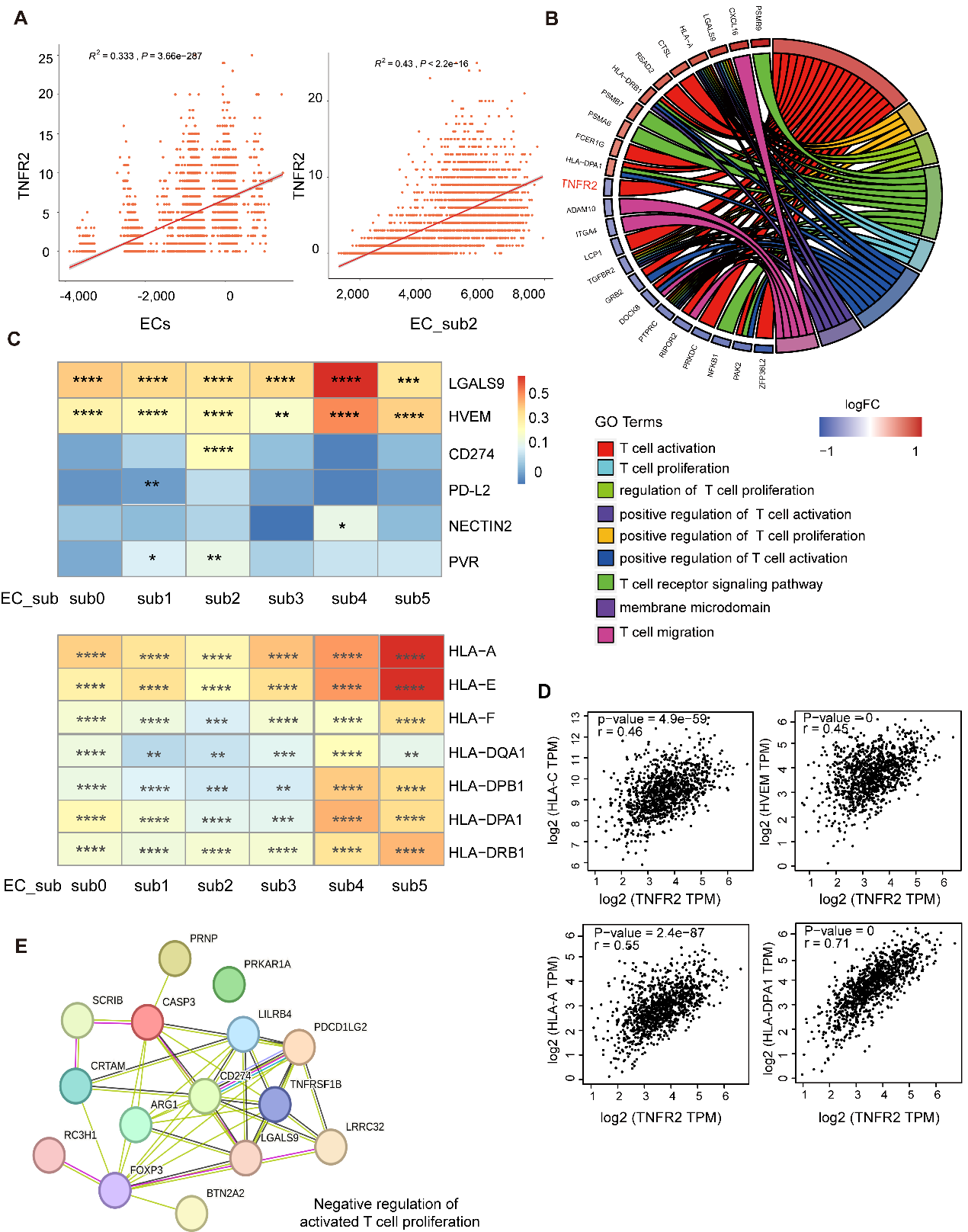


**Fig. S6** The crucial role of TNFR2 in endothelial immunosuppression. **A,** The correlation between either the whole ECs (left) or EC_sub2 (right) and TNFR2 from scRNA-seq dataset. **B,** Circular plot of GO analysis showing T cell-related pathways of marker genes in EC_sub2. **C,** Relationship between TNFR2 and immune checkpoints (upper) or MHC molecules (blow) across EC_subclusters. Color indicates correction intensity. Color scale: red, strong correction; blue, weak correction. **D,** GEPIA2-based Spearman correlation analysis showing pertinence of *TNFR2* to representative EC ligands in breast cancer tissue. **E,** A network of protein-protein interaction revealing the correction of TNFR2 with molecules involved in GO term “negative regulation of activated T cell proliferation”. *P < 0.05, **P < 0.01, ***P < 0.001, ****P < 0.0001.


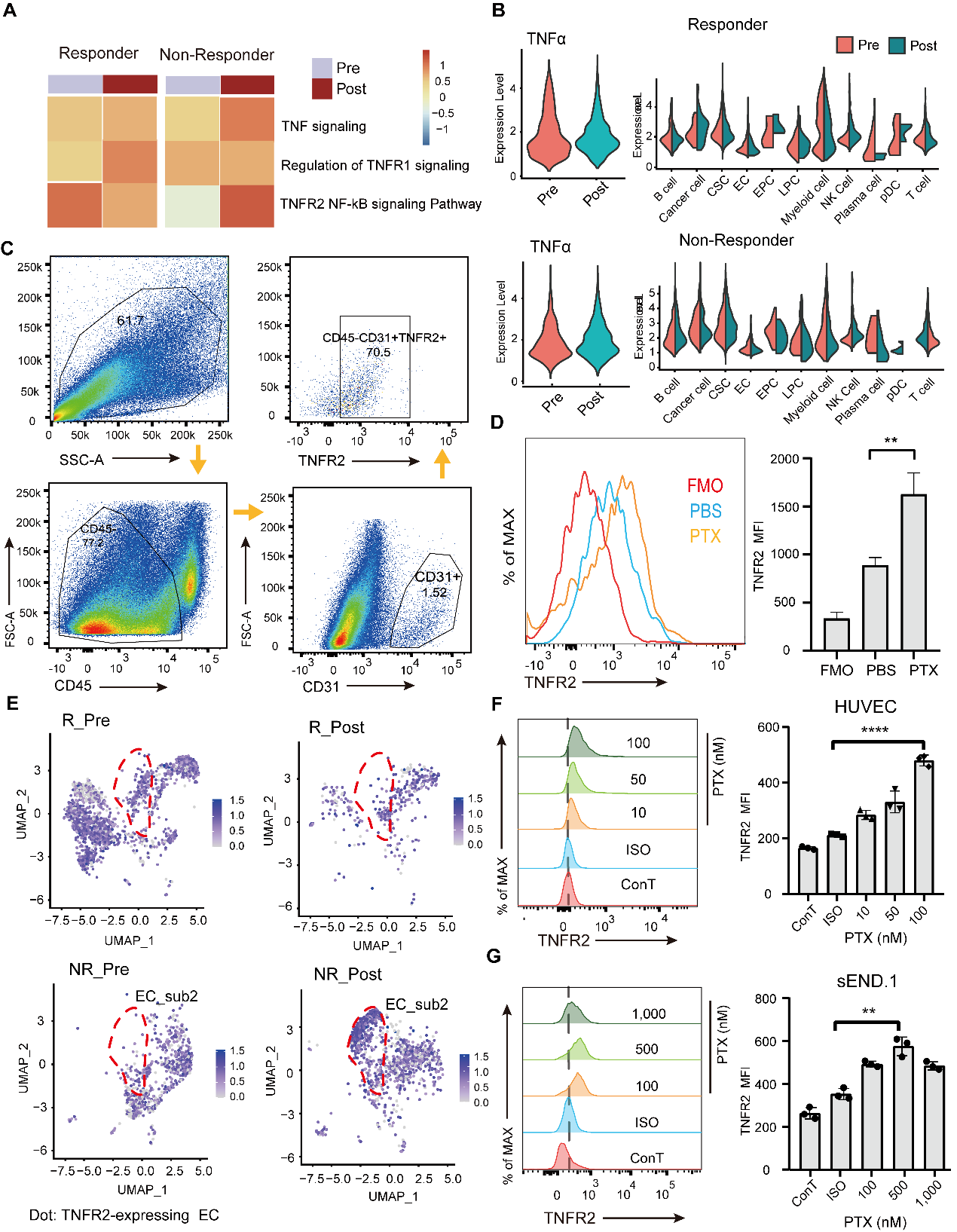


**Fig. S7** Increased TNFR2+ ECs upon PTX treatment. **A,** Differentially activated TNF/TNFR-related pathways in EC_sub2. **B,** Expression levels of *TNF* in the whole (left) and individual cell type (right). **C,** Gating scheme for TNFR2+ ECs (CD45−CD31+TNFR2+) from tumor tissues. **D,** Representative histograms (left) and quantification (right) of TNFR2 expression on ECs isolated from 4T1–transplanted mice 26day after implantation. FMO serves as a negative control. **E,** UMAP plot of TNFR2-expressing ECs. **F,** Stimulative effect of PTX on TNFR2 expression on HUVEC was tested by flow cytometry; n = 3. **G,** Another sort of EC, sEND.1 was tested for TNFR2 expression following PTX treatment; n = 3. **P < 0.01, ****P < 0.0001.

Abbreviation: FACS, fluorescence activated cell sorting; MFI, mean fluorescence intensity; FMO, Fluorescence minus one.


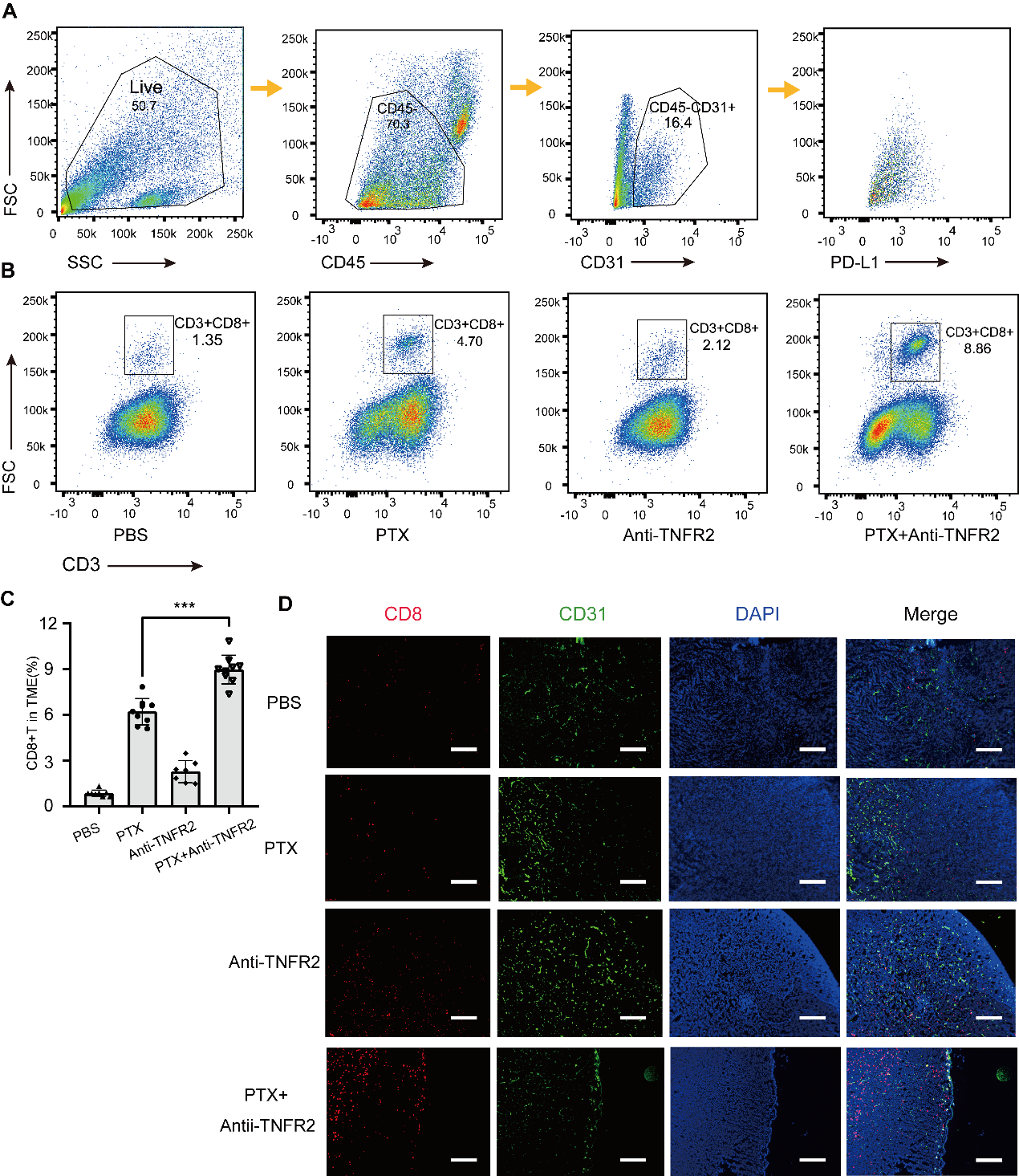


**Fig. S8** Augmented CD8+T cell infiltration into tumors following pharmacologically targeting TNFR2. **A,** Gating scheme for PD-L1+ ECs (CD45−CD31+PD-L1+) isolated from tumors. **B,** Flow plots of CD8+T cell; values indicating the proportion of CD3+CD8+T cell. **C,** Quantification of the proportion of CD8+T cells. **D,** IHC staining for CD8 (red) and CD31 (green) in representative tumor sections. Scale bar: 200 µm. ***P < 0.001.
